# The clinical and molecular landscape of diffuse hemispheric glioma, H3 G34-mutant

**DOI:** 10.1093/neuonc/noaf015

**Published:** 2025-01-22

**Authors:** Emilie Le Rhun, Andrea Bink, Joerg Felsberg, Dorothee Gramatzki, Sebastian Brandner, Jamal K Benhamida, Antje Wick, Joerg C Tonn, Malte Mohme, Ghazaleh Tabatabai, David Capper, Matija Snuderl, Evangelia Razis, Michael W Ronellenfitsch, Nicolas Neidert, Ho-Keung Ng, Ute Pohl, Tejus Bale, Stefanie Quach, David Rieger, Ulrich Schüller, Julia Onken, Katharina Drüschler, Claude-Alain Maurage, Luca Regli, Estelle Healy, Maya Graham, Tibor Hortobagyi, Simon Paine, Leslie Bridges, Tereza Lausova, Valentina Medici, Philipp Sievers, David Schrimpf, Wolfgang Wick, Felix Sahm, Guido Reifenberger, Andreas von Deimling, Michael Weller

**Affiliations:** Department of Medical Oncology and Hematology, University Hospital Zurich, Zurich, Switzerland; Department of Neurology, University Hospital and University of Zurich, Zurich, Switzerland; Department of Neurosurgery, Clinical Neuroscience Center, University Hospital and University of Zurich, Zurich, Switzerland; Department of Neuroradiology, Clinical Neuroscience Center, University Hospital and University of Zurich, Zurich, Switzerland; German Cancer Consortium (DKTK), Partner Site Essen/Düsseldorf, Düsseldorf, Germany; Institute of Neuropathology, Medical Faculty, Heinrich Heine University and University Hospital Düsseldorf, Düsseldorf, Germany; Department of Neurology, University Hospital and University of Zurich, Zurich, Switzerland; Department of Neurodegenerative Disease, Queen Square Institute of Neurology, University College London, London, UK; Division of Neuropathology, National Hospital for Neurology and Neurosurgery, University College London NHS Foundation Trust, London, UK; Department of Pathology and Laboratory Medicine, Memorial Sloan Kettering Cancer Center, New York, New York, USA; Department of Neurology and Neurooncology Program, National Center for Tumor Diseases, University Hospital Heidelberg, Heidelberg, Germany; Clinical Cooperation Unit Neurooncology, German Cancer Research Center (DKFZ), Heidelberg, Germany; German Consortium for Translational Cancer Research (DKTK), PartnerSite Munich, Munich, Germany; Department of Neurosurgery, University Hospital, LMU Munich, Munich, Germany; Department of Neurosurgery, University Medical Center Hamburg-Eppendorf, Hamburg, Germany; Center for Neuro-Oncology, Comprehensive Cancer Center Tübingen-Stuttgart, Eberhard Karls University Tübingen, Tübingen, Germany; German Consortium for Translational Cancer Research (DKTK), Partner Site Tübingen, Tübingen, Germany; Cluster of Excellence (EXC 2180) “Image Guided and Functionally Instructed Tumor Therapies,” Eberhard Karls University Tübingen, Tübingen, Germany; Department of Neurology & Interdisciplinary Neuro-Oncology, University Hospital Tübingen, Hertie Institute for Clinical Brain Research, Eberhard Karls University Tübingen, Tübingen, Germany; German Cancer Consortium (DKTK), Partner Site Berlin, German Cancer Research Center (DKFZ), Heidelberg, Germany; Department of Neuropathology, Charité - Universitätsmedizin Berlin, corporate member of Freie Universität Berlin and Humboldt-Universität zu Berlin, Berlin, Germany; Department of Pathology, Molecular Pathology and Diagnostics, NYU Langone Medical Center, New York, New York, USA; Department of Oncology, Hygeia Hospital, Athens, Greece; University Cancer Center (UCT), University Hospital Frankfurt, Goethe University, Frankfurt am Main, Germany; Dr. Senckenberg Institute of Neurooncology, University Hospital Frankfurt, Goethe University, Frankfurt am Main, Germany; Department of Neurosurgery, Medical Center, University of Freiburg, Freiburg, Germany; Department of Anatomical and Cellular Pathology, Chinese University of Hong Kong, Hong Kong, Hong Kong; Department of Histopathology, Department of Cellular Pathology, University Hospital Birmingham, Birmingham, UK; Department of Pathology and Laboratory Medicine, Memorial Sloan Kettering Cancer Center, New York, New York, USA; Department of Neurosurgery (Evangelisches Klinikum Bethel), Medical School, Bielefeld University, Bielefeld, Germany; German Consortium for Translational Cancer Research (DKTK), PartnerSite Munich, Munich, Germany; Department of Neurosurgery, University Hospital, LMU Munich, Munich, Germany; Department of Neurology & Interdisciplinary Neuro-Oncology, University Hospital Tübingen, Hertie Institute for Clinical Brain Research, Eberhard Karls University Tübingen, Tübingen, Germany; Research Institute Children’s Cancer Center Hamburg, Hamburg, Germany; Department of Pediatric Hematology and Oncology, Research Institute Children’s Cancer Center Hamburg, University Medical Center Hamburg-Eppendorf, Hamburg, Germany; Institute of Neuropathology, University Medical Center Hamburg-Eppendorf, Hamburg, Germany; Humboldt-University, Berlin, Germany; Department of Neurosurgery, Charité University Medicine Berlin, Berlin, Germany; German Cancer Consortium (DKTK), Partner Site Berlin, German Cancer Research Center (DKFZ), Heidelberg, Germany; Department of Neurology and Neurooncology Program, National Center for Tumor Diseases, University Hospital Heidelberg, Heidelberg, Germany; Clinical Cooperation Unit Neurooncology, German Cancer Research Center (DKFZ), Heidelberg, Germany; Department of Pathology, Centre Biologie Pathologie, Lille University Hospital, Hopital Nord, Lille, France; Department of Neurosurgery, Clinical Neuroscience Center, University Hospital and University of Zurich, Zurich, Switzerland; Department of Pathology, Royal Hospitals, Belfast, Northern Ireland; Department of Neurology, Memorial Sloan Kettering Cancer Center, New York, New York, USA; Department of Neuropathology, University Hospital Zurich, Zurich, Switzerland; Department of Cellular Pathology, Queen’s Medical Centre Campus, Nottingham, UK; Department of Cellular Pathology, St George’s University Hospitals NHS Foundation Trust St George’s Hospital, London, UK; Clinical Cooperation Unit Neuropathology, German Consortium for Translational Cancer Research (DKTK), German Cancer Research Center (DKFZ), Heidelberg, Germany; Department of Neuropathology, Institute of Pathology, University Hospital Heidelberg, Heidelberg, Germany; Department of Neuroradiology, Clinical Neuroscience Center, University Hospital and University of Zurich, Zurich, Switzerland; Clinical Cooperation Unit Neuropathology, German Consortium for Translational Cancer Research (DKTK), German Cancer Research Center (DKFZ), Heidelberg, Germany; Department of Neuropathology, Institute of Pathology, University Hospital Heidelberg, Heidelberg, Germany; Clinical Cooperation Unit Neuropathology, German Consortium for Translational Cancer Research (DKTK), German Cancer Research Center (DKFZ), Heidelberg, Germany; Department of Neuropathology, Institute of Pathology, University Hospital Heidelberg, Heidelberg, Germany; Department of Neurology and Neurooncology Program, National Center for Tumor Diseases, University Hospital Heidelberg, Heidelberg, Germany; Clinical Cooperation Unit Neurooncology, German Cancer Research Center (DKFZ), Heidelberg, Germany; Clinical Cooperation Unit Neuropathology, German Consortium for Translational Cancer Research (DKTK), German Cancer Research Center (DKFZ), Heidelberg, Germany; Department of Neuropathology, Institute of Pathology, University Hospital Heidelberg, Heidelberg, Germany; German Cancer Consortium (DKTK), Partner Site Essen/Düsseldorf, Düsseldorf, Germany; Institute of Neuropathology, Medical Faculty, Heinrich Heine University and University Hospital Düsseldorf, Düsseldorf, Germany; Clinical Cooperation Unit Neuropathology, German Consortium for Translational Cancer Research (DKTK), German Cancer Research Center (DKFZ), Heidelberg, Germany; Department of Neuropathology, Institute of Pathology, University Hospital Heidelberg, Heidelberg, Germany; Department of Neurology, University Hospital and University of Zurich, Zurich, Switzerland

**Keywords:** glioblastoma, histone, loss, methylation, *MGMT*

## Abstract

**Background:**

Diffuse hemispheric glioma, histone 3 (H3) G34-mutant, has been newly defined in the 2021 World Health Organization (WHO) classification of central nervous system tumors. Here we sought to define the prognostic roles of clinical, neuroimaging, pathological, and molecular features of these tumors.

**Methods:**

We retrospectively assembled a cohort of 114 patients (median age 22 years) with diffuse hemispheric glioma, H3 G34-mutant, central nervous system WHO grade 4, and profiled the imaging, histological, and molecular landscape of their tumors.

**Results:**

Compared with glioblastoma, H3 G34-mutant diffuse hemispheric gliomas exhibited less avid contrast enhancement, necrosis, and edema on MRI. Comprehensive analyses of mutational and DNA copy number profiles revealed recurrent mutations in *TP53* and *ATRX*, homozygous deletions of *CDKN2A/B*, and amplifications of *PDGFRA*, *EGFR*, *CCND2*, and *MYCN*. *MGMT* promoter methylation was detected in 79 tumors (75%); 11 tumors (13%) showed DNA copy number profiles suggestive of circumscribed deletions on 10q26.3 involving the *MGMT* locus. Median survival was 21.5 months. Female sex, gross total resection, and *MGMT* promoter methylation were positive prognostic factors on univariate analysis. Among radiological, pathological, and molecular features, the absence of pial invasion and the presence of microvascular proliferation and *CDK6* amplification were positive prognostic factors on univariate analyses.

**Conclusions:**

This study refines the clinical and molecular landscape of H3 G34-mutant diffuse hemispheric gliomas. Dedicated trials for this novel tumor type are urgently needed.

**Key Points**

- We define clinical disease and molecular characteristics of a novel tumor type.
- We delineate neuroimaging features that may aid in recognizing this tumor type.
- Female sex, gross total resection, and *MGMT* promoter methylation are positive prognostic factors.

**Importance of the Study**

This study sought to generate a profile for patient, clinical disease, and molecular characteristics of a novel, molecularly defined tumor entity: diffuse hemispheric glioma, H3 G34-mutant.

We delineate neuroimaging features that may aid in recognizing this entity and to distinguish it from glioblastoma. We characterize the molecular landscape of these tumors beyond the canonical H3 G34 driver mutation and identify female sex, gross total resection, and *MGMT* promoter methylation as positive prognostic factors.

Diffuse hemispheric glioma, histone H3 G34-mutant, central nervous system (CNS) World Health Organization (WHO) grade 4, has been newly defined as a distinct tumor type among the pediatric-type diffuse high-grade gliomas in the 2021 World Health Organization (WHO) Classification of Tumors of the Central Nervous System (CNS).^1^ These tumors were previously considered as part of the spectrum of isocitrate dehydrogenase (IDH)-wildtype glioblastomas,^2^ but have been separated because of their characteristic molecular profile and their preferential occurrence in children, adolescents, and young adults.^3^ They are defined by missense mutations in the *H3-3A* gene including c.103G>A p.Gly35Arg (G34R), c.103G>C p.Gly35Arg (G34R), and c.104G>T p.Gly35Val (G34V)^1,4,5^ and display a distinct DNA methylome profile.^4,6,7^ Frequent alterations in addition to H3 G34 mutations are mutations of *ATRX* and *TP53*, *PDGFRA* mutation or amplification, *CDKN2A/B* homozygous deletion, amplification of *CDK4*, *CDK6* or *CCND2*, and *MGMT* promoter methylation.^3,4,8–13^ Certain DNA copy number alterations, in particular losses of chromosome arms 3q and 4q, have been detected more commonly in diffuse hemispheric gliomas, H3 G34-mutant, than in IDH-wildtype glioblastoma.^6^ Histologically, H3 G34-mutant diffuse hemispheric gliomas may present with a broad spectrum of divergent features with microscopic characteristics ranging from those typical of IDH-wildtype glioblastoma to those resembling embryonal CNS tumors with primitive neuronal features, formerly referred to as primitive neuroectodermal tumors (PNETs).^6,14^

No specific neuroimaging features have been identified that reliably distinguish H3 G34-mutant diffuse hemispheric gliomas from adult types of high-grade diffuse gliomas, notably IDH-wildtype glioblastoma.^15–18^ As H3 G34-mutant diffuse hemispheric gliomas are rare tumors, data on patient outcomes are limited to small patient cohorts. A larger study in the pediatric patient population indicated longer survival of children with H3 G34-mutant diffuse hemispheric glioma compared to children with H3 K27-altered diffuse midline glioma or other IDH-wildtype and H3-wildtype pediatric-type diffuse high-grade gliomas.^8^ Another study reported that adult patients with H3 G34-mutant diffuse hemispheric glioma showed a similarly poor survival as patients with H3 K27-altered diffuse midline glioma or IDH-wildtype glioblastoma.^10^ According to a meta-analysis of 27 studies (135 patients), H3 G34-mutant diffuse hemispheric glioma appears to be associated with overall poor outcome as indicated by median time to progression of 10.0 months, median time from progression to death of 5.0 months, and median overall survival of 17.3 months. Favorable prognostic factors were age above 18 years and near or gross total resection.^3^ Molecular factors that have been associated with inferior prognosis of H3 G34-mutant diffuse hemispheric glioma patients include G34V rather than G34R type of *H3-3A* mutation, presence of *PDGFRA* or *EGFR* amplification, and lack of *MGMT* promoter methylation.^6,11^

Here, we report on the clinicopathological characteristics and comprehensive molecular characterization of a multi-institutional, retrospectively assembled cohort of 114 patients with H3 G34-mutant diffuse hemispheric glioma. We confirm an overall poor prognosis for these patients, but delineate a characteristic profile of clinical, histological, and molecular parameters that may facilitate recognition of this probably underdiagnosed tumor and may aid in the development of interventional trials for patients with this particular glioma type.

## Methods

### Patients

Clinical data, imaging data, pathological reports including molecular testing results, and tissue specimens as available of patients from the participating centers were centrally collected in Zurich, Switzerland. Patients were registered either by clinical Neuro-Oncology centers mainly involved in adult patient care or by Departments of Neuropathology. A local diagnosis of an H3 G34-mutant diffuse hemispheric glioma based on the demonstration of an H3 G34 missense mutation by immunohistochemistry or DNA sequencing, or by DNA methylation profiling demonstrating the respective methylome profile was required for inclusion in this study.

### Ethics Statements

The sponsor of the study was the University Hospital Zurich. The Cantonal Ethics Committee of the Canton of Zurich approved the project (2022-00521). The control cohort study (reference cohorts 1 and 2) was also approved by the Cantonal Ethics Committee of the Canton of Zurich (KEK-ZH 2009-0135/1, KEK-ZH 2015-0437) (Note S1). The German Glioma Network (GGN) (reference cohort 3) was a prospective, noninterventional cohort study that involved 8 clinical centers in Germany (www.gliomnetzwerk.de) and was supported by the German Cancer Aid from 2004 to 2012.

### Neuroimaging

Central neuroradiology review of the diagnostic MRI of 40 cases from the H3 G34 cohort and 50 cases from a second reference cohort with IDH-wildtype glioblastoma (“reference cohort 2”) was performed at the Department of Neuroradiology, University Hospital Zurich by A.B. and V.M., using an adaptation of the Visually AcceSAble Rembrandt Images approach (https://wiki.nci.nih.gov/display/CIP/VASARI) (Supplementary Table S1). The sequences for central review included T2-weighted, fluid-attenuated inversion recovery (FLAIR), T1-weighted pre- and postcontrast sequences, diffusion-weighted imaging, and apparent diffusion coefficient maps.

### Central Pathology Review

We collected 102 tumor specimens from the 114 patients with H3 G34-mutant diffuse hemispheric glioma. In total, tumors from 89 patients were subjected to central pathology review at the Brain Tumor Reference Center of the German Society for Neuropathology and Neuroanatomy (DGNN), Institute of Neuropathology, Heinrich Heine University Düsseldorf (G.R., J.F.). The histological review was based on microscopical investigation of hematoxylin-eosin (H&E)-stained sections that were available from 64 tumors (51 patients) or on evaluation of high-resolution whole-slide digital scans of H&E-stained sections that were available for 38 tumors (38 patients) or both. For 25 patients, neither microscopical sections nor digital scans were available for central review. For these patients, classification relied on the local diagnoses and the molecular findings obtained by *H3-3A* mutation analysis and DNA methylome profiling (see below). Tumors for which glass slides or digital scans were available were centrally confirmed as high-grade diffuse astrocytic gliomas and evaluated for the following morphological features: tumor cell density (low, moderate, high), pleomorphism (low, moderate, high), presence of a small cell PNET-like component (yes, no), mitotic rate (number of mitoses per 10 high power fields at 400× magnification corresponding to 2.37 mm^2^), presence of necrosis (yes, no), presence of pathologic microvascular proliferation (yes, no), presence of necrosis and/or microvascular proliferation (yes, no), presence of multinucleated giant cells (yes, single, no), and presence of calcifications (yes, no). All tumors were classified as diffuse hemispheric gliomas, H3 G34-mutant based on the integration of histologic and molecular features as recommended in the fifth edition of the WHO classification of tumors of the CNS,^1^ with molecular features corresponding to demonstration of an H3 G34 missense mutation or an assignment to the corresponding DNA methylation class of the Heidelberg brain tumor classifier or both.

### Mutation Analysis of *H3-3A* and DNA Methylome Profiling

The type of H3 G34 mutation was determined either at the local center or upon central pathology review using immunohistochemistry with mutation-specific antibodies against H3 G34R (clone RM240) or H3 G34V (clone R307, RevMAb Biosciences), Sanger sequencing, droplet digital PCR of the *H3-3A* mutation hotspot,^19^ or gene panel sequencing. Tumors from 84 patients were subjected to DNA methylome analysis using Illumina 850k (EPIC) or Illumina 450k DNA methylation arrays (Illumina). DNA methylome analyses were performed at the participating centers or upon central pathology review, and data were evaluated centrally at the Institute of Neuropathology, University of Heidelberg (A.v.D.) based on the Heidelberg brain tumor classifier version v12.8 complemented with classifier version v11b.4 when the prediction score was below 0.90.^7^

### Copy Number Profiles and tSNE Analyses

DNA copy number profiles were calculated from the DNA methylation datasets by subjecting raw data to the “conumee” R package (https://github.com/mwsill/conumee).^20^ Copy number alterations were assessed post additional baseline corrections. Amplifications were called if the respective probes exhibited a value higher than 0.5 on a log2 scale. Homozygous deletions were called if the respective probes exhibited a value lower than −0.5 on a log2 scale. tSNE analyses were conducted using the R-Package Rtsne (https://github.com/jkrijthe/Rtsne) employing the 20,000 most variable CpG sites according to standard deviation; 3000 iterations and a perplexity value of 10. The overall copy number variation (CNV) load of combined gains and losses was determined employing a proprietary algorithm.^21^

### *MGMT* Promoter Methylation Status and Chromosomal Representation

The *MGMT* promoter methylation status was determined based on the DNA methylome data sets using the *MGMT*-STP27 model.^7,22^ For comparative analyses of the DNA methylome data, reference cohort 3 of gliomas from the GGN was also analyzed, including IDH-wildtype glioblastoma subclass mesenchymal (*N* = 20), subclass receptor tyrosine kinase (RTK)1 (*N* = 20), subclass RTK2 (*N* = 20), and IDH-mutant glioma, subclass high-grade astrocytoma (*N* = 20), subclass astrocytoma (*N* = 20), and subclass 1p/19q-codeleted oligodendroglioma (*N* = 20).^23^

The copy number status of *MGMT* in diffuse hemispheric gliomas, H3 G34-mutant was assessed as described above utilizing 2 threshold levels. Cases designated with an underrepresentation exhibiting a value lower than −0.5 (less stringent) or −0.75 (more stringent) on a log2 scale. *MGMT* copy number representation in diffuse hemispheric gliomas, H3 G34-mutant was compared to those in a GGN control cohort of 120 gliomas. In addition, *MGMT* copy number representation in diffuse hemispheric gliomas, H3 G34-mutant was compared to that of 2267 RTK1, 3924 RTK2, and 2267 mesenchymal glioblastomas, IDH-wildtype analyzed in a recent study.^24^

### Gene Panel Sequencing

Thirty-eight diffuse hemispheric gliomas, H3 G34-mutant were also subjected to gene panel next-generation sequencing covering the entire coding and selected intronic and promoter regions of 130 genes recurrently altered in brain tumors^25^ (Note S2). For comparative analysis of *TP53* mutation profiles, we selected 116 representative high-grade glioma samples from the Heidelberg database. These samples were filtered based on a methylation class family score greater than 0.8, as determined by the brain tumor classifier v12.8. The selected samples comprised H3 K27-altered diffuse midline glioma (*n* = 57), as well as the IDH-wildtype glioblastoma subtypes glioblastoma (GBM) MES (*n* = 27), GBM RTK1 (*n* = 22), and GBM RTK2 (*n* = 10).^7^ Data visualization was created using ProteinPaint.^26^

### Statistical Analyses

Demographic, clinical, and molecular data are presented with descriptive statistics. The Chi-square test was performed for the analysis of nominal variables, and the Mann-Whitney U test was used for the comparison of ordinal variables between groups. Progression-free survival was defined as the time between the date of diagnostic surgery and the date of first progression. Overall survival was defined as the time between the date of diagnostic surgery and the date of death. Kaplan-Meier curves were compared using the log-rank test. Patients without an event were censored at the date of the last follow-up before the database lock. Patients of the control cohort were also censored at the last follow-up. Median follow-up for the whole patient cohort was estimated using the reversed Kaplan-Meier method. Univariate and multivariate analyses were done using Cox regression. The multivariate model was applied to all patients who had complete information on all tested covariables, that is, neither the missing data imputation technique was applied, nor a correction for multiple comparisons. For statistical analysis, SPSS Version 29 was used (SPSS IBM Corp.), and a *P* value of.05 was considered statistically significant.

## Results

### Patient Characteristics

A cohort of 114 patients with diffuse hemispheric glioma, H3-G34-mutant was assembled. Patients were diagnosed from 2005 to 2022, with 2 additional patients diagnosed in 1997, and were registered by 30 different institutions. DNA sequencing and/or immunohistochemistry with mutant-specific antibodies detected the following histone 3 missense mutations: p.G35R (G34R) in 85 patients, p.G35V (G34V) in four patients, and p.G35M (G34M) in one patient. Information on the specific mutation type was not available for 24 patients, including 20 patients in whom the diagnosis was based on DNA methylation profiling alone plus 4 cases where the type of mutation was not specified by the submitting institution (Supplementary Figure S1A). After completion of the central review of all tissues made available, 89 were H3 G34R-mutant, 8 were H3 G34V-mutant, and 13 tumors remained that were diagnosed based on methylation profiling alone (Supplementary Figure S1B). For 3 patients, the diagnosis was based on local sequencing, but the type of mutation was not provided. A hitherto undescribed *H3-3A* p.Gly35Met (G34M) missense variant was detected in a 14-year-old male patient with a tumor that also exhibited *ATRX* and *TP53* mutations. The patient received chemoradiotherapy but died after 13 months. One H3 G34R-mutant diffuse hemispheric glioma additionally displayed an IDH1 p.R132C variant by sequencing.

Clinical disease characteristics of the 114 patients included in this study are summarized in Table 1. The median age at diagnosis was 22 years (range 8-70 years), 76 patients (67%) were male, and 38 patients (33%) were female. Compared with a population-based cohort of patients with IDH-wildtype glioblastoma from the Canton of Zurich, Switzerland,^27^ that was re-classified according to the 2021 WHO classification (reference cohort 1, Note S1),^1^ patients with diffuse hemispheric glioma, H3 G34-mutant were younger (*P* < .001), had a higher Karnofsky performance status at diagnosis (*P* < .001), had a biopsy at diagnosis more often by trend (*P* = .067), and their tumors exhibited *MGMT* promoter methylation more often (*P* < .001). These disease characteristics were similar in the 13 patients that were diagnosed based on methylation profiling alone (Supplementary Table S1).

**Table 1.** H3 G34-mutant diffuse hemispheric glioma: patient and tumor characteristics.

	Diffuse hemispheric glioma, H3 G34 mutant *n* = 114	Glioblastoma, IDH-wildtype^a^ *n* = 352	*P* value
**Age at first surgery**			
Median (years)	22; 25^b^	66	<.001; <.001^b^
Interquartile interval (Q1-Q3)	18-30	58-73	
Range (minimum-maximum)	8-70; 18-70^b^	29-90	-
**Sex, *n* (%)**			
Male	76 (67)	222 (63)	.487
Female	38 (33)	130 (37)	
**Age, *n* (%)**			
<18 years	26 (23)	-	<.001
18-49 years	85 (75)	34 (10)	
50-59 years	2 (2)	65 (19)	
60-69 years	1 (1)	127 (36)	
≥ 70 years	0 (0)	126 (36)	
**KPS at diagnosis, *n* (%)**			
90-100%	46 (52)	49 (14)	<.001
70-80%	28 (32)	205 (59)	
<70%	14 (16)	94 (27)	
No data	26	4	
**Extent of resection, *n* (%)**			
Gross total resection	38 (36)	113 (32)	.067
Incomplete	33 (31)	154 (44)	
Biopsy	36 (34)	83 (24)	
Autopsy	0 (0)	1 (0.3)	
No data	7	1	-
**IDH mutation status (local assessment by sequencing or methylation profiling), *n* (%)**			
Mutant	1 (1)	-	-
Wildtype	91 (99)	352 (100)	-
No data	22	0	-
***MGMT* promoter methylation status, *n* (%)**			
Methylated	79 (75)	104 (44)	<.001
Unmethylated	26 (25)	130 (56)	
No data	10	118	-
**First-line treatment, *n* (%)**			
No therapy	7 (7)	62 (18)	.004^c^
Any therapy	100 (94)	284 (82)	
Radiotherapy alone	3 (3)	80 (23)	<.001^d^
Temozolomide alone	3 (3)	25 (7)	
Temozolomide/radiotherapy	7 (7)	20 (6)	
Temozolomide/radiotherapy, followed by temozolomide	70 (65)	116 (34)	
Temozolomide/radiotherapy, followed by temozolomide-based regimen	13 (12)^e^	11 (3)^f^	
Radiotherapy followed by temozolomide	1 (1)	17 (5)	
Surgery alone with no planned adjuvant treatment	1 (1)	0 (0)	
Other	2 (2)^g^	15 (4)^h^	
No data	7	6	-
**Radiotherapy, *n* (%)**			
Yes	96 (90)	258 (75)	<.001
Information on time interval, *n* (%)^i^	91 (94.8)	255 (99)	-
Information on dose, *n* (%)^i^	85 (88.5)	199 (77)	-
No	11 (10.3)	88 (25)	
No data	7	6	
**Time interval between surgery and radiotherapy, *n* (%)**			
Median (days)	31	29	.137
Interquartile interval (Q1-Q3)	25-39	23-38	
Range (days)	12-95	0-91	-
**Dose of radiotherapy, *n* (%)**			
Median (Gy)	60	60	<.001
Interquartile interval (Q1-Q3)	60-60	40-60	
Range (Gy)	13-62	5-60	-
**Maintenance temozolomide, *n* (%)**			
Yes	88 (83)	169 (50)	<.001
Information on the number of cycles, *n* (%)^k^	76 (86)	155 (9192)	-
No	18 (17)	177 (51)	
No data	8	6	-
**Number of cycles of temozolomide-based maintenance therapy**			
Median	6	5	.002
Interquartile interval (Q1-Q3)	3-8	2-6	
Range	1-20	1-16	-
**First-line treatment completed as planned, *n* (%)**			
Yes	37 (43)	123 (46)	.546
No	50 (58)	143 (54)	
No first-line treatment	7	62	-
No data	16	24	-
Ongoing	4	0	
**Outcome**			
Median follow-up of surviving patients (months, 95% CI)	14.1 (11.5-16.7)	5.2 (3.8-6.7)	.001
Median follow-up (reverse Kaplan-Meier)	114.4 (31.0-197.7)	40.2 (18.1-62.4)	.001
Events (progression)	79	318	-
No progression during follow-up, *n*	4	34	
No data on progression-free survival, *n*	31	-	-
Median progression-free survival (months, 95 % CI)	9.7 (7.2-12.3)	4.7 (4.3-5.1)	< .001
Events (death)	71	312	-
No data on overall survival, *n*	2	-	-
Alive or lost to follow-up at the time of the analysis, *n*	41	40	-
Median overall survival (months, 95% CI)	21.5 (15.3-27.7)	11.3 (9.7-13.0)	<.001

^a^Reference cohort from the Cancer Registry Zurich 2005-2014,^27,35^ including only patients aged 18 years or more.

^b^Excluding patients younger than 18 years.

^c^Between no therapy and any therapy.

^d^Between no therapy and the different treatment options.

^e^Temozolomide/radiotherapy→temozolomide plus lomustine (CeTeG) (*n* = 6); temozolomide/radiotherapy-based study (*n* = 7).

^f^Temozolomide/radiotherapy-based study (*n* = 7); temozolomide/radiotherapy→temozolomide plus TTF (*n* = 4).

^g^Carboplatin plus VP16, then radiotherapy then temozolomide (initial diagnosis of medulloblastoma) (*n* = 1), temozolomide/radiotherapy→unknown (*n* = 1).

^h^Radiotherapy plus bevacizumab (*n* = 12), lomustine alone (*n* = 1), radiotherapy plus experimental agent study (*n* = 2), radiotherapy plus temozolomide plus bevacizumab (*n* = 1).

^i^Percentages calculated for the patients who had radiotherapy.

^k^Percentages calculated for the patients who had maintenance temozolomide.

IDH, isocitrate dehydrogenase; MGMT, O^6^-methylguanine DNA methyltransferase.

### Neuroimaging Features

**Figure 1.**
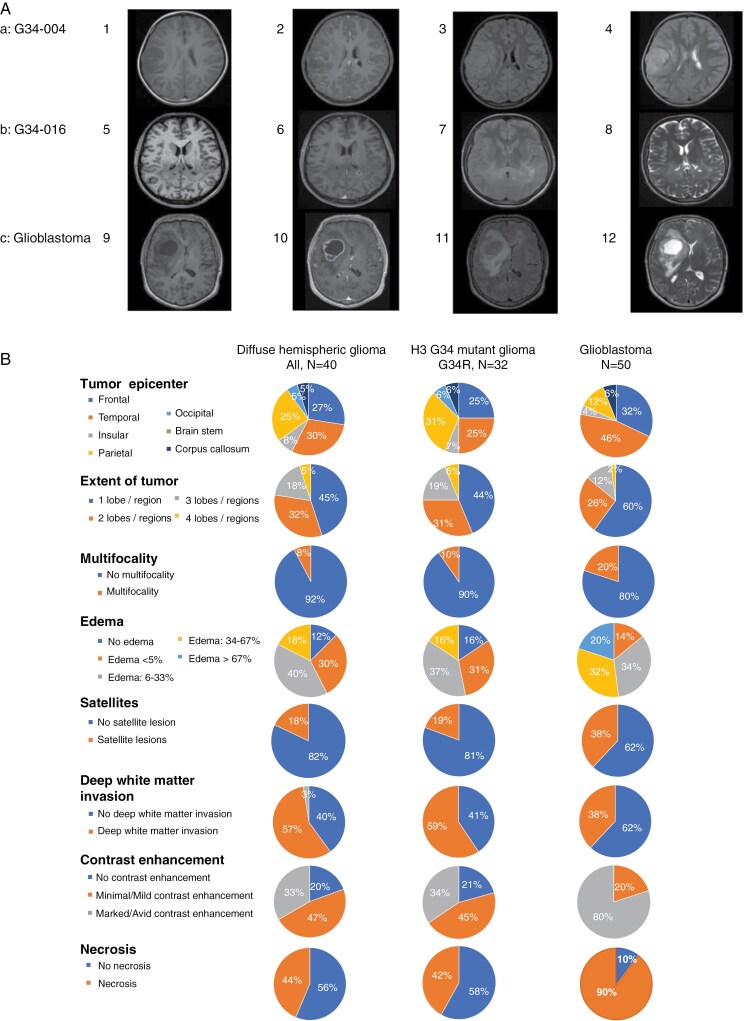
**Typical MRI features of G34-mutant glioma.** (A) Axial sequences (1, 5, 9: T1-weighted sequences without contrast, 2,6,10: contrast-enhanced T1-weighted sequences, 3, 7, 11: FLAIR sequences; 4, 8,12: T2-weighted sequences). 1-4: 8-year-old patient with an intra-axial frontal H3 G34V-mutant diffuse hemispheric glioma with involvement of the cortex and white matter, calvarial remodeling, mild contrast enhancement, minimal necrosis, and relatively small edema. 5-8: 28-year-old patient with an H3 G34R-mutant extensive intra-axial diffuse hemispheric glioma extending via the forceps major into both hemispheres with edema, small areas of necrosis, and minimal contrast enhancement. 9-12: 60-year-old patient with an intra-axial left-sided fronto-insular tumor with peripheral marked enhancement, extensive necrosis, and edema, histologically diagnosed as glioblastoma, IDH-wildtype. (B) Neuroimaging features of G34-mutant diffuse hemispheric glioma: comparison with glioblastoma, IDH-wildtype. IDH, isocitrate dehydrogenase.

MRI data of 40 patients with diffuse hemispheric gliomas, H3 G34-mutant prior to the first surgical intervention were available for central radiology review (Supplementary Tables S2 and S3). Characteristic imaging features are shown in Figure 1A. Tumors were extensive with more than one CNS region involved in 22 patients (55%) but rarely multifocal (3 patients, 8%). Edema was absent in 5 patients (13%) and below 5% of the entire tumor volume in 12 patients (30%). No patient had severe edema (>67% of the entire tumor volume), but a mass effect was noted in 36 patients (90%). Pial invasion was noted in 30 patients (77%), and ependymal extension in 21 patients (53%). We also noted that 7 tumors (19%) did not exhibit contrast enhancement, and 17 further tumors (47%) had only minimal or mild contrast enhancement. Necrosis was absent in 22 patients (56%). Hemorrhage was noted in 12 patients (31%). Calvarial remodeling was observed in 7 patients (18%).

Compared with a reference cohort of 50 patients with IDH-wildtype glioblastoma (reference cohort 2), diffuse hemispheric gliomas, H3 G34-mutant were characterized by less edema (*P* < .001), fewer satellite lesions (*P* = .039), smaller tumor size (*P* = .013), less avid contrast enhancement (*P* < .001), lower frequency of necrosis (*P* < .001), but numerically more frequent parietal location (*P* = .239) and deep white matter invasion (*P* = .038) (Supplementary Table S3, Figure 1B). Of note, calvarial remodeling was observed only in the H3-G34 cohort.

### Neuropathology

Central pathology review was performed in 89 of the 114 H3 G34-mutant diffuse hemispheric glioma patients (78%) from either the initial operation (*n* = 86) or a recurrent operation (*n* = 3) when tissue from initial surgery was not available. All tumors evaluated by central pathology review were confirmed as diffuse high-grade astrocytic gliomas, with the absence of necrosis and/or microvascular proliferation in 30 patients (34%) but the presence of necrosis or microvascular proliferation or both in 59 patients (66%) among the diagnostic samples (Supplementary Table S4, Supplementary Figure S2). In total, 61 tumors (69%) showed solely an astrocytic differentiation, whereas 28 tumors (31%) demonstrated a small cell/PNET-like phenotype or at least a PNET-like component. A mitotic count of ≥10 mitoses per 10 microscopical high power fields (corresponding to 2.37 mm^2^) was noted in 38 tumors (51%). Areas of necrosis were present in 40 tumors (45%) and microvascular proliferation in 53 samples (60%). The tumors of patients with necrosis or contrast enhancement on MRI more frequently showed histological necrosis or vascular proliferation (Supplementary Table S5).

### DNA Methylation Profiling and Copy Number Variation Analysis

**Figure 2.**
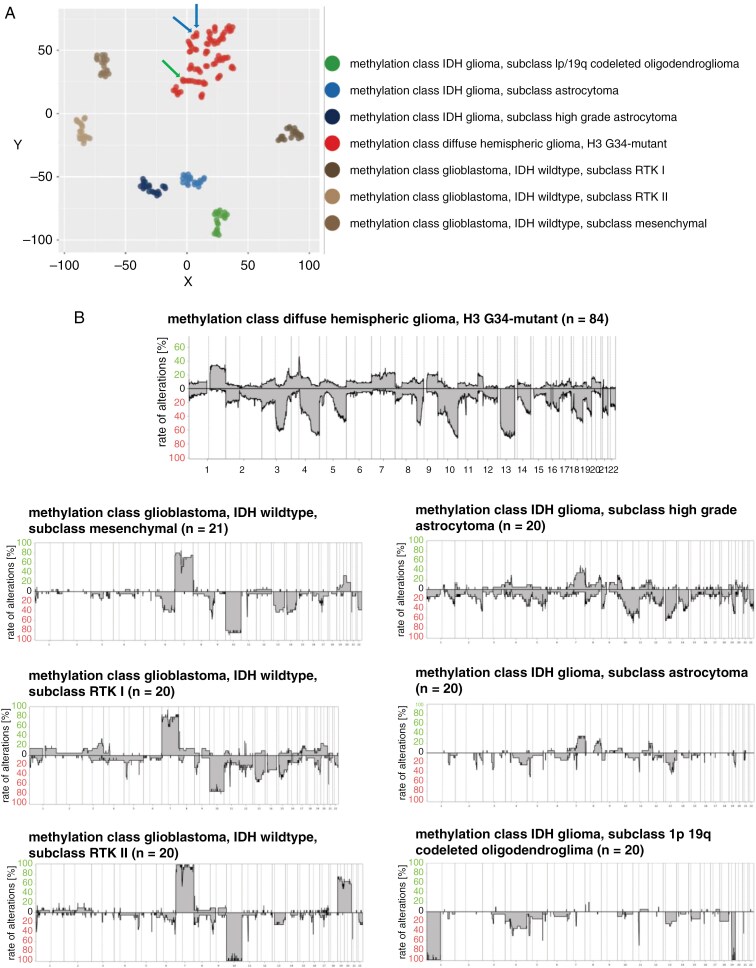
**Molecular characterization of diffuse hemispheric glioma, H3 G34-mutant. (**A) tSNE plot in distinction to other types of gliomas in adulthood from the GGN cohort, including IDH-wildtype glioblastoma subclass mesenchymal (*N* = 20), subclass RTK1 (*N* = 20), subclass RTK2 (*N* = 20), and IDH-mutant glioma, subclass high-grade astrocytoma (*N* = 20), subclass astrocytoma (*N* = 20), and subclass 1p/19q-codeleted oligodendroglioma (*N* = 20)^23^ (blue arrows: tumors with typical H3 G34 methylation profile but without H3 G34 mutation identified by local analysis or central gene panel sequencing; green arrow: the single H3 G34-mutant tumor with an IDH mutation confirmed by local analysis and central gene panel sequencing. DNA methylation profiling was done on 65 newly diagnosed tumors, 6 recurrent tumors, and 13 patients where this information was lacking, for overall 84 patients (74%). (B) CNV profiles in distinction to the other types of gliomas as in A. GGH, German Glioma Network; IDH, isocitrate dehydrogenase; RTK, receptor tyrosine kinase.

DNA methylation profiling was done in 84 diffuse hemispheric gliomas with H3 G34 mutation (74%). Two cases with centrally confirmed H3 G34 mutation did not exhibit a typical methylation profile for G34-mutant gliomas: one was assigned to the methylation class of IDH-wildtype mesenchymal glioblastoma (score v12.8, 0.86) and one did not receive a reliable prediction. These were male patients aged 54 and 17 years at diagnosis who received temozolomide chemoradiotherapy and who survived for 13 and 26 months. The other 82 tumors exhibited the typical methylation profile of diffuse hemispheric glioma, H3 G34-mutant, including the single tumor with an IDH mutation, and 2 tumors with typical methylation profile, but without canonical mutation (Figure 2A). The v12.8 prediction score for diffuse hemispheric glioma, H3 G34-mutant was 0.91 or higher in 71 cases. Among the 11 remaining tumors, the v11.4 classifier also diagnosed H3 G34-mutant gliomas with prediction scores above 0.91 for 5 cases. Homozygous losses of *CDKN2A/B* or *RB1* were seen in subgroups of patients, with homozygous *RB1* deletions being enriched in H3 G34-mutant diffuse hemispheric gliomas when compared to IDH-wildtype glioblastomas and IDH-mutant gliomas from the GGN cohort (reference cohort 3) (Supplementary Table S6). Recurrent gene amplifications were noted for *PDGFRA*, *CDK6, CCND2*, *EGFR*, and *MYCN*. *PDGFRA* and *CDK6* amplification were enriched in diffuse hemispheric gliomas when compared to other adult-type gliomas (Supplementary Table S6). Copy number variation analysis showed that the H3 G34-mutant tumors resembled IDH-wildtype glioblastomas with regard to chromosome 10 loss, whereas chromosome 7 gain was not common (Figure 2B). Only 14 H3 G34-mutant tumors (17%) exhibited a + 7/-10 copy number alteration, whereas 68 tumors (83%) did not.

Given the limited power when conducting too many formal comparisons, we refrained from doing so when exploring the DNA copy number profiles. We noted no specific clustering of H3 G34R- versus H3 G34V-mutant tumors by their DNA copy number profiles, but more frequent chromosome 3 and 13 losses in H3 G34V-mutant tumors (Supplementary Figure S3). Tumors of females appeared to have more often 9q gains and 10q losses than tumors of males (Supplementary Figure S4). There were no specific CNV patterns by age (Supplementary Figure S5). More gains of chromosome 4 were noted in tumors that were topographically not restricted to one lobe, but otherwise, no specific CNV profile by primary tumor location was identified (Supplementary Figure S6). There was a trend for more frequent 1q gains in tumors with more marked contrast enhancement (Supplementary Figure S7), but no association with diffusion abnormalities (Supplementary Figure S8). Tumors with imaging necrosis showed generally more CNV alterations, notably losses on chromosomes 10, 13, and 18 and gains on 20; 1p was often lost in tumors with necrosis, but frequently gained in tumors without necrosis (Supplementary Figure S9). Regarding histological differentiation, the comparison of tumors with PNET-like components (*n* = 22) versus tumors with astrocytic histology (*n* = 49) indicated preferential 4q and 5q losses in tumors with PNET-like components (Supplementary Figure S10). Histological evidence of necrosis was not associated with specific CNV alterations (Supplementary Figure S11). Overall, chromosome arm 10q was balanced in 18 tumors, lost in 17 tumors, and exhibited segmental changes in 47 tumors. At a threshold of −0.5 (log2), focal copy number underrepresentations on distal 10q suggestive of biallelic losses affecting the *MGMT* locus were noted in 21 samples, 16 of 61 tumors with a methylated *MGMT* promoter and 5 of 20 tumors with an unmethylated *MGMT* promoter (Supplementary Figure S12A). At a more stringent threshold of −0.75 (log2), such changes at the *MGMT* locus were noted in 11 samples, 9 of 61 tumors with a methylated *MGMT* promoter and 2 of 20 tumors with an unmethylated *MGMT* promoter. Representative CNV profiles of these tumors are depicted in Supplementary Figure S12B. To estimate the frequency of such circumscribed copy number losses affecting *MGMT* in H3 G34-mutant gliomas compared with glioblastoma, we analyzed the *MGMT* locus not only in our reference cohort 3 (Figure 2), but also in another extensive cohort of patients with IDH-wildtype glioblastoma.^24^ At the threshold of −0.75 (log2), focal copy number underrepresentations affecting the *MGMT* locus were seen more frequently in H3 G34-mutant gliomas (13%) here than in mesenchymal (MES) (0.3%), RTK1 (4%), or RTK2 (6%) glioblastoma in a large reference cohort (Supplementary Table S7).

### Gene Panel Sequencing

**Figure 3.**
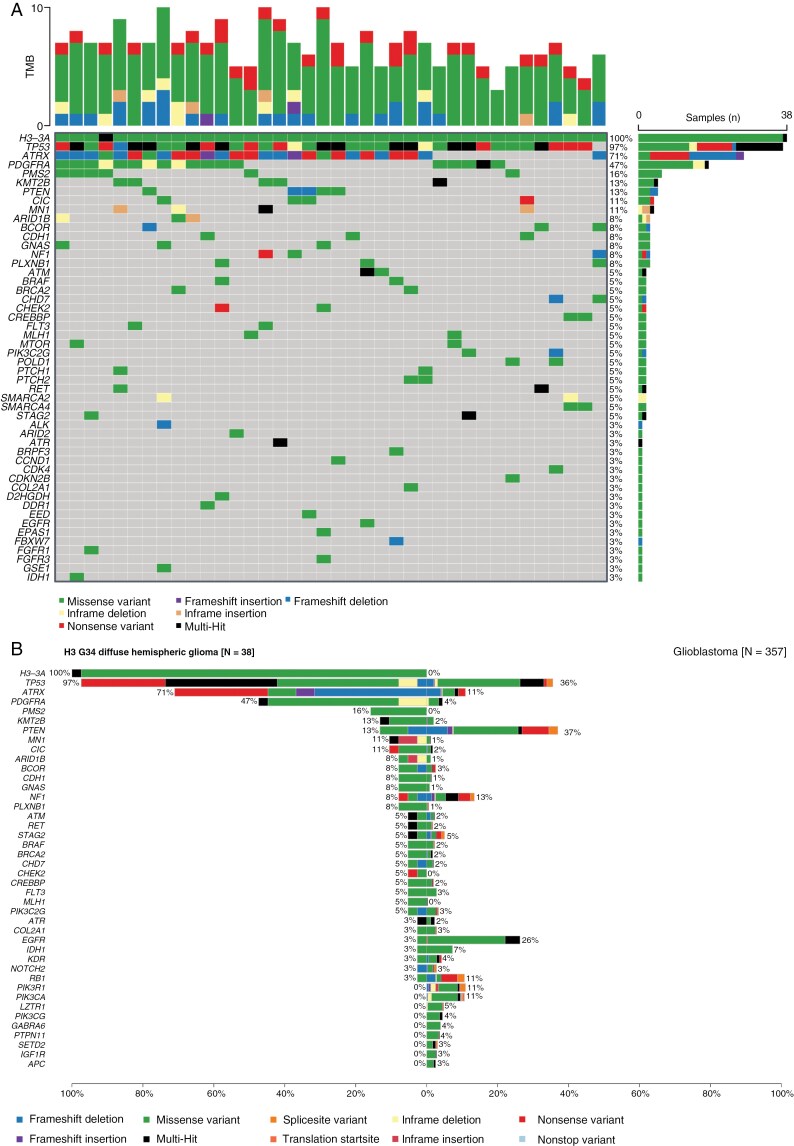
**Mutational landscape of diffuse hemispheric gliomas, H3 G34-mutant. (**A) Mutational landscape of diffuse hemispheric glioma, H3 G34-mutant. The tumor mutational burden (TMB) part shows the total number of mutations per megabase. (B) CoBarplot of H3 G34-mutant gliomas (left) compared with glioblastoma, IDH-wildtype (right). IDH, IDH, isocitrate dehydrogenase.

Gene panel sequencing covering 130 CNS tumor-associated genes^25^ was performed for diffuse hemispheric gliomas, H3 G34-mutant of 38 patients (72%), including 37 newly diagnosed and 1 recurrent tumor. In one case, no H3 G34 mutation was identified by sequencing; however, methylation profiling and CNV plot were typical of H3 G34-mutant glioma, with a score of 0.99. The most commonly mutated genes besides *H3-3A* were *ATRX*, *TP53*, and *PDGFRA*; furthermore, recurrent mutations in *PMS2* and *KMT2B* were noted (Figure 3A). There were no clear differences in the mutational landscape by type of *H3-3A* mutation although *PDGFRA* alterations were relatively more common in tumors carrying the H3 G34V mutation (*P* = .100) (Supplementary Figure S13). Compared with IDH-wildtype glioblastoma, we noted higher frequencies of *TP53* (*P* < .001), *ATRX* (*P* < .001), and *PDGFRA* (*P* < .001) mutations, and lower frequencies of alterations notably in *PTEN* (*P* = .003), *NF1* (*P* = .375), and *EGFR* (*P* = .001) (Figure 3B). Interestingly, the types of *TP53* mutations detected in H3 G34-mutant diffuse hemispheric gliomas were different from those typically observed in diffuse midline glioma, H3 K27-altered or glioblastoma, IDH-wildtype. There was a broad distribution of different *TP53* mutations in diffuse hemispheric gliomas, H3 G34-mutant with only a slight enrichment of the hotspot p.R273C mutation (Supplementary Figure S14).

### Treatment and Outcome

**Figure 4.**
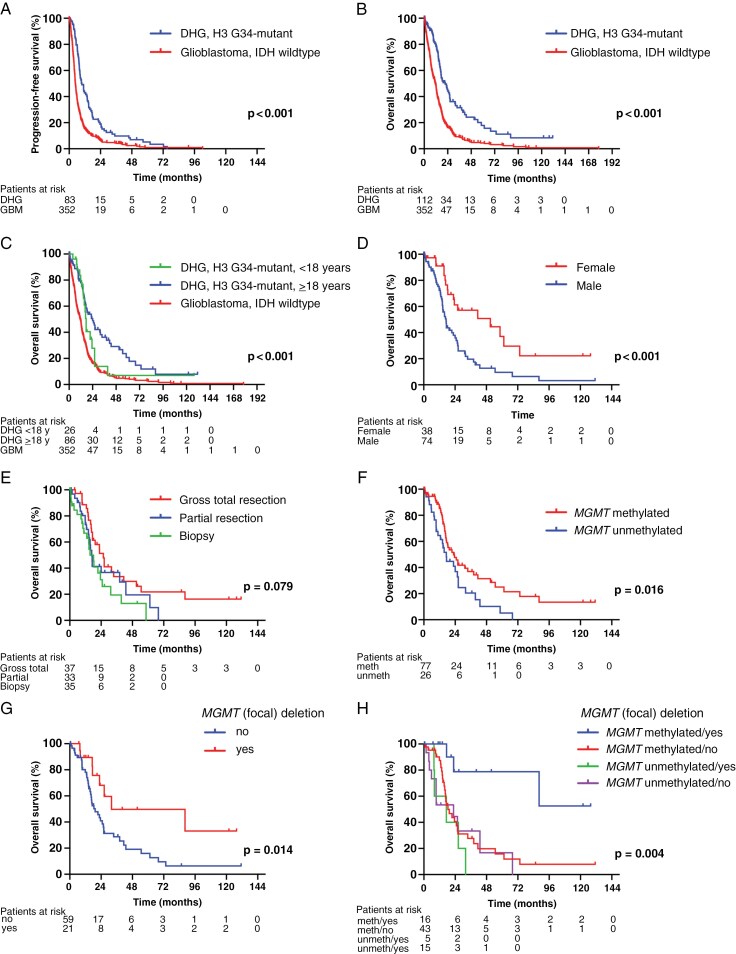
**Survival analyses in patients with diffuse hemispheric glioma, H3 G34-mutant.** Progression-free (A) and overall survival (B) are depicted by Kaplan-Meier curves. (C) Overall survival with diffuse hemispheric glioma, G34-mutant, split by age. A-C include data on the reference cohort of patients with glioblastoma, IDH-wildtype, E-H contain data on H3 G34-mutant diffuse hemispheric glioma. Survival associations with sex (D) and extent of resection (E). Survival associations by *MGMT* promoter methylation (F) and by focal underrepresentation of chromosome 10 including the *MGMT* locus (cutoff: −0.5) (G) stratified by *MGMT* promoter methylation status (H). The log-rank test was used for comparison, and a *P* value of.05 was defined as statistically significant. IDH, isocitrate dehydrogenase; MGMT, O^6^-methylguanine DNA methyltransferase.

Initial treatment of the patients with H3 G34-mutant diffuse hemispheric glioma is summarized in Table 1 and SupplementaryTable S8. According to local documentation, the surgical resection was gross total for 38 patients (36%), partial for 33 patients (31%), and 36 patients (34%) had a biopsy only. A total of 91 patients (80%) had a combination of radiotherapy and temozolomide as the first-line treatment. The median follow-up for the entire cohort was 40.2 months; it was 14.1 months for the surviving patients (*N* = 41, alive at the last follow-up). Median progression-free survival was 9.7 months (95% confidence interval [CI] 7.2-12.3) compared to 4.7 months (95% CI 4.3-5.1) (*P* < .001) for IDH-wildtype glioblastoma patients in the population-based reference cohort 1. Median overall survival was 21.5 months (95% CI 15.3-27.7) for patients with H3 G34-mutant diffuse hemispheric gliomas compared to 11.3 months (95% CI 9.7-13.0; *P* < .001) for IDH-wildtype glioblastoma patients in the reference cohort 1; yet, survival was poor in pediatric patients with H3 G34-mutant diffuse hemispheric glioma (Figure 4C). There were no obvious differences in the disease characteristics to explain this observation (Supplementary Table S9). Eight of 112 patients (7%) with sufficient follow-up information (7%) were alive at 5 years. These long-term survivors were more often female, had more often received a gross total resection, and their tumors exhibited *MGMT* promoter methylation more often than patients with confirmed death prior to 5 years, but CNV plots looked similar (Supplementary Table S10, Supplementary Figure S15).

### Prognostic Factors

Univariate analysis identified female sex (hazard ratio [HR]=0.40, 95% CI 0.23-0.69), gross total resection (HR=0.52, 95% CI 0.29-0.93, biopsy reference), and methylated *MGMT* promoter status (HR=0.52, 95% CI 0.30–0.89) as associated with improved overall survival; female sex and gross total resection remained significant in the multivariate analysis (Figure 4, Table 2). A similar analysis conducted for progression-free survival confirmed sex and *MGMT* promoter status, but not the extent of resection on univariate analysis, and identified the extent of resection and *MGMT* promoter status, but not female sex, on multivariate analysis (Supplementary Table S11). As a sensitivity analysis, we conducted a similar analysis focused on the population of 72 patients with a diagnostic confirmation of H3 G34-mutant diffuse hemispheric glioma by sequencing and methylation profiling. This analysis confirmed sex and *MGMT* promoter status, but not the extent of resection on univariate analysis, and sex, but not the extent of resection or *MGMT* promoter status, on multivariate analysis (Supplementary Table S12). Univariate analysis of patients younger than 18 years (*N* = 26) revealed that female sex (*N* = 7) was not associated with superior overall survival (HR=0.58, 95% CI 0.21-1.67, *P* = .312), whereas methylated *MGMT* promoter status (*N* = 15) was confirmed to be associated with better survival (HR=0.28, 95% CI 0.89-0.88, *P* = .029).

**Table 2.** Univariate and multivariate analysis of prognostic factors for death in patients with diffuse hemispheric glioma, H3 G34 mutant (Cox regression).

		Univariate analysis				Multivariate analysis	
	*n* (events)	HR (95 % CI)		*P* value	*n* (events)	HR (95 % CI)	*P* value
**Age**							
<18 years	26 (18)	1.42 (0.82-2.44)		.212	13 (9)	1.58 (0.71-3.52)	.261
≥18 years	86 (53)	1		ref	61 (38)	1	ref
**Sex**							
Female	38 (17)	0.40 (0.23-0.69)		.001	31 (12)	0.43 (0.21-0.87)	.019
Male	74 (54)	1		ref	43 (35)	1	ref
**KPS**							
<70%	14 (9)	1.92 (0.90-4.08)		.092	11 (8)	1.05 (0.32-3.45)	.937
70-80%	27 (17)	1.20 (0.66-2.20)		.552	24 (15)	0.94 (0.43-2.06)	.879
90-100%	45 (29)	1		ref	39 (24)	1	ref
No data	26	-		-	-		
**Extent of resection**							
Gross total	37 (25)	0.52 (0.29-0.93)		.028	28 (19)	0.31 (0.12-0.79)	.014
Incomplete	33 (21)	0.77 (0.42-1.40)		.384	22 (15)	0.40 (0.16-1.05)	.062
Biopsy/Autopsy	35 (22)	1		ref	24 (13)	1	ref
No data	7	-		-	-		
***MGMT* promoter status**							
Methylated	77 (43)	0.52 (0.30-0.89)		.018	53 (29)	0.51 (0.23-1.13)	.098
Unmethylated	26 (20)	1		ref	21 (18)	1	ref
No data	9	-		-	-		
**First-line treatment**							
No therapy	6 (4)	1		ref	4 (4)	1	ref
Any therapy	99 (63)	0.02 (0.03-0.70)		<.001	70 (43)	0.27 (0.03-0.14)	<.001
No data	7	-	-		-		

Survival data are missing in *N* = 2 patients.

CI, confidence interval; HR, hazard ratio; KPS, Karnofsky performance status; MGMT, O^6^-methylguanine DNA methyltransferase.

Univariate analysis of radiographical features identified only pial invasion as a negative prognostic factor (Supplementary Table S13). On univariate analyses of the type of H3 G34 mutation and pathological features, microvascular proliferation (HR=0.53, 95% CI 0.31-0.91, *P* = .020) was associated with a better prognosis (Supplementary Table S14). No associations with CNV profiles became apparent when looking at short versus intermediate versus long survival in tertiales, including censoring at the last follow-up (Supplementary Figure S16). When only confirmed deaths were considered, losses of chromosomes 8 and 12q were associated with shorter survival (Supplementary Figure S17). Among the common molecular alterations, *CDK6* amplification was associated with superior outcome, whereas the +7/−10 signature and amplification of other genes were not associated with survival (Supplementary Table S15). The overall mutational load of combined gains and losses was also not prognostic (data not shown). Patients with a focal underrepresentation of chromosome 10 at the *MGMT* locus detected by EPIC array analysis had longer survival than patients without this copy number change (*P* = .014 for cutoff −0.5, *P* = .057 for cutoff −0.75), and this difference was driven by patients with tumors with *MGMT* promoter methylation (Figure 4, Supplementary Tables S15-S18). In fact, the positive prognostic association of *MGMT* promoter methylation was essentially related to the superior outcome of patients with tumors with *MGMT* promoter methylation and focal losses at the *MGMT* locus.

## Discussion

Diffuse hemispheric glioma, H3 G34-mutant, has been defined as a distinct tumor type among the category of pediatric-type diffuse high-grade gliomas in the 2021 WHO classification.^1^ H3 G34 mutations are driver mutations that are thought to cause tumorigenesis by an epigenetic mechanism altering gene expression. H3 G34-mutant tumors are probably initiated by the histone 3 mutation but additionally carry frequent genetic alterations in other genes including *ATRX*, *TP53*, *PDGFRA*, *EGFR*, *CDKN2A*, and others.^3,4,8–13^ Half of the tumors exhibit activating *PDGFRA* mutations or *PDGFRA* gene amplification that are selected for at tumor recurrence; furthermore, the H3 G34 mutation may no longer be required for tumor growth, whereas mutant PDGFRA may drive recurrence,^28^ suggesting that it may represent a druggable target for therapeutic intervention.

The present study is so far the most extensive effort at defining disease characteristics of H3 G34-mutant diffuse hemispheric glioma with a focus on the adult patient population. We have identified female sex as a prognostic factor that has not emerged in the pediatric population. We provide guidance for imaging features that should alert to the differential diagnosis of H3 G34-mutant diffuse hemispheric glioma, which may be of particular importance in resource-restricted situations where genetic H3 G34 mutation testing or DNA methylation profiling are not routinely performed. The relative frequency of H3 G34 mutations in the pediatric as opposed to the adult population reported here is likely underestimated since our consortium includes many centers mainly involved in adult brain tumor patient care. Since population-based incidence data on this tumor type are not available, we cannot estimate the relative frequency of H3 G34-mutant diffuse hemispheric glioma, for example, compared with glioblastoma, IDH-wildtype.

There is interest in differential phenotypes conferred by different types of H3 G34 mutation, both at the cellular level^29^ as well as at the level of patient outcome.^11^ We found only minor differences in the CNV (Supplementary Figure S3) and mutational profiles (Supplementary Figure S13) in H3 G34R- versus H3 G34V-mutant tumors. In addition, we did not observe a prognostic role of the H3 G34R versus H3 G34V mutation (*P* = .457). Furthermore, we found 2 patients with a typical methylation profile of H3 G34-mutant glioma without the canonical mutation (Figure 2A). Such patients may have mutations in the related *H3-3B* gene,^30^ but this was excluded in 2 of these patients by panel sequencing.

*PDGFRA* amplification has been reported to be more common in diffuse hemispheric gliomas, H3 G34-mutant with glioblastoma-like histology versus tumors with primitive neuronal/neuroectodermal tumor-like features, while *CCND2* amplification showed the opposite trend,^6^ but this was not seen in our cohort (Note S3). Potential future targets for intervention in subgroups of patients include *PDGFRA* mutation or amplification, *CDKN2A/B* deletion, or amplification of *CDK4*, *CDK6,* or *CCND2* (Figure 3A, Supplementary Table S6). In fact, CDK6 has recently been reported as a vulnerability in interneuron lineage progenitors which have been proposed to give rise H3 G34-mutant diffuse hemispheric glioma.^31^ In contrast to a large pediatric study that reported frequent *FBXW7* mutations,^32^ we observed only one *FBXW7* mutation, in a 17-year old patient, suggesting a link of this mutation to pediatric tumors. Furthermore, we report less frequent variants in the DNA mismatch repair gene, *PMS2*, and in the *KMT2B* gene (Figure 3A). *KMT2B* mutations have so far been mainly linked to movement disorders^33^ and whether the variants found here are pathogenic or represent variants of unknown significance remains to be clarified.

In the absence of dedicated clinical trials, current treatment algorithms propose to treat patients with diffuse hemispheric glioma, H3 G34-mutant, with temozolomide-based chemoradiotherapy since *MGMT* promoter methylation is frequent in these tumors.^34^ Given the retrospective and uncontrolled nature of this study, we cannot derive meaningful conclusions regarding clinical management. However, efforts at gross total resection should be encouraged, and the high rate of *MGMT* promoter methylation and also the focal copy number underrepresentation affecting the *MGMT* locus in a fraction of patients with *MGMT* promoter-unmethylated tumors suggest that alkylating agent chemotherapy should be part of the first-line treatment in this disease.

Overall, the outcome of H3 G34-mutant glioma patients is poor. It was overall better here than for patients with IDH-wildtype glioblastoma (Figure 4), but there may be no difference when comparing these 2 tumor types in patient groups matched for prognostic factors, notably age and *MGMT* promoter methylation status.

Limitations of our study include its retrospective nature, the lack of standardized treatment and follow-up, the lack of systematic exclusion of H3 G34 mutations in the reference cohort 1, the lack of a population-based approach, and the small sample size for patients with long-term survival. However, studies like this report currently represent the best starting point to learn more about this new tumor type, so as to aid the standardization of diagnosis and treatment and to facilitate the design and conduct of successful clinical trials. The retrospective and prospective EORTC 2013 GLIO-RARE project (NCT05259605) serves this purpose.

In conclusion, this study provides a clinical, neuroimaging, histological, and molecular genetic characterization of H3 G34-mutant diffuse hemispheric gliomas. Novel targeted therapeutic interventions are awaited, but it seems prudent not to exclude patients with H3 G34-mutant gliomas at least from early glioblastoma trials as long as no dedicated trials for these tumors are available.

## Supplementary Material

noaf015_suppl_Supplementary_Material

## Data Availability

Coded data not provided in the article will be made available upon reasonable request and after approval from centers for purposes of replicating results. Requests for further information and resources should be directed to the corresponding author: Emilie Le Rhun (emilie.lerhun@usz.ch).
