# Supplementary material for: The clinical and molecular landscape of diffuse hemispheric glioma, H3 G34-mutant
Source: Neuro Oncol. 2025 Jan 22;27(6):1519–35. doi: 10.1093/neuonc/noaf015 (PMC12309718; doi:10.1093/neuonc/noaf015)
Supplement: noaf015_suppl_Supplementary_Material [file noaf015_suppl_supplementary_material.docx]

**SUPPLEMENTARY MATERIAL**

**The clinical and molecular landscape of diffuse hemispheric glioma, H3 G34-mutant**

Emilie Le Rhun, MD, Andrea Bink, MD, Joerg Felsberg, MD, Dorothee Gramatzki, MD, Sebastian Brandner, MD, Jamal K Benhamida, MD, Antje Wick, MD, Joerg C. Tonn, MD, Malte Mohme, MD, Ghazaleh Tabatabai, MD, David Capper, MD, Matija Snuderl, MD, Evangelia Razis, MD, Michael W. Ronellenfitsch, MD, Nicolas Neidert, MD, Ho-Keung Ng, MD, Ute Pohl, MD, Tejus Bale, MD, Stefanie Quach, MD, David Rieger, MD, Ulrich Schüller, MD, Julia Onken, MD, Katharina Drüschler, MD, Claude-Alain Maurage, MD, Luca Regli, MD, Estelle Healy, MD, Maya Graham, MD, Tibor Hortobagyi, FRCPath, Simon Paine, MD, Leslie Bridges, MD, Tereza Lausova, MD, Valentina Medici, MD, Philipp Sievers, MD, David Schrimpf, Wolfgang Wick, MD, Felix Sahm, MD, Guido Reifenberger*, MD, Andreas von Deimling*, MD , Michael Weller*, MD, for the H3 G34 DHG Study Group

(1) Department of Neurosurgery, Clinical Neuroscience Center, University Hospital and University of Zurich, Switzerland (ELR, LR)

(2) Department of Neurology, University Hospital and University of Zurich, Switzerland (ELR, DG, MW)

(3) Department of Medical Oncology and Hematology, University Hospital Zurich, Switzerland (ELR)

(4) Department of Neuroradiology, Clinical Neuroscience Center, University Hospital and University of Zurich, Switzerland (AB, VM)

(5) Institute of Neuropathology, Medical Faculty, Heinrich Heine University and University Hospital Düsseldorf, Düsseldorf, Germany (JF, GR)

(6) German Cancer Consortium (DKTK), partner site Essen/Düsseldorf, Düsseldorf, Germany (JF, GR)

(7) Division of Neuropathology, National Hospital for Neurology and Neurosurgery, University College London NHS Foundation Trust, London, United Kingdom (SB)

(8) Department of Neurodegenerative Disease, Queen Square Institute of Neurology, University College London, London, United Kingdom (SB)

(9) Department of Pathology and Laboratory Medicine, Memorial Sloan Kettering Cancer Center, New York, United States (JKB, TB)

(10) Clinical Cooperation Unit Neurooncology, German Cancer Research Center (DKFZ), Heidelberg, Germany (AW, KD, WW)

(11) Department of Neurology and Neurooncology Program, National Center for Tumor Diseases, University Hospital Heidelberg, Heidelberg, Germany (AW, KD, WW)

(12) Department of Neurosurgery, University Hospital, LMU Munich, Munich, Germany, and German Consortium for Translational Cancer Research (DKTK), Partner Site Munich, Germany (JCT, SQ)

(13) Department of Neurosurgery, University Medical Center Hamburg-Eppendorf, Hamburg, Germany (MM)

(14) Department of Neurology & Interdisciplinary Neuro-Oncology, University Hospital Tübingen, Hertie Institute for Clinical Brain Research, Eberhard Karls University Tübingen, Germany (GT, DR)

(15) Cluster of Excellence (EXC 2180) "Image Guided and Functionally Instructed Tumor Therapies", Eberhard Karls University Tübingen, 72076, Tübingen, Germany (GT)

(16) German Consortium for Translational Cancer Research (DKTK), Partner Site Tübingen, 72076, Tübingen, Germany (GT)

(17) Center for Neuro-Oncology, Comprehensive Cancer Center Tübingen-Stuttgart, Eberhard Karls University Tübingen, 72076, Tübingen, Germany (GT)

(18) Department of Neuropathology, Charité - Universitätsmedizin Berlin, corporate member of Freie Universität Berlin and Humboldt-Universität zu Berlin, Berlin, Germany (DC)

(19) German Cancer Consortium (DKTK), Partner Site Berlin, German Cancer Research Center (DKFZ), Heidelberg, Germany (DC, JO)

(20) Department of Pathology, Molecular Pathology and Diagnostics, NYU Langone Medical Center, New York, United States (MS)

(21) Department of Oncology, Hygeia Hospital, Athens, Greece (ER)

(22) Dr. Senckenberg Institute of Neurooncology, University Hospital Frankfurt, Goethe University, Frankfurt am Main, Germany (MWR)

(23) University Cancer Center (UCT), University Hospital Frankfurt, Goethe University, Frankfurt am Main, Germany (MWR)

(24) Department of Neurosurgery, Medical Center, University of Freiburg, Freiburg, Germany (NN)

(25) Department of Anatomical and Cellular Pathology, Chinese University of Hong Kong, Hong Kong (HKN)

(26) Department of Histopathology, Department of Cellular Pathology, University Hospital Birmingham, United Kingdom (UP)

(27) Department of Neurosurgery (Evangelisches Klinikum Bethel), Medical School, Bielefeld University, Bielefeld, Germany (SQ)

(28) Institute of Neuropathology, University Medical Center Hamburg-Eppendorf, Hamburg, Germany (US)

(29) Department of Pediatric Hematology and Oncology, Research Institute Children's Cancer Center Hamburg, University Medical Center Hamburg-Eppendorf, Hamburg, Germany (US)

(30) Research Institute Children's Cancer Center Hamburg, Hamburg, Germany (US)

(31) Department of Neurosurgery, Charité University Medicine Berlin, Berlin, Germany (JO)

(32) Humboldt-University, Berlin, Germany (JO)

(33) Department of Pathology, Centre Biologie Pathologie, Lille University Hospital, Hopital Nord, Lille, France (CAM)

(34) Department of Pathology, Royal Hospitals, Belfast, Northern Ireland (EH)

(35) Department of Neurology, Memorial Sloan Kettering Cancer Center, New York, United States (MG)

(36) Department of Neuropathology, University Hospital Zurich, Switzerland (TH)

(37) Department of Cellular Pathology, Queen’s Medical Centre Campus, Nottingham, United Kingdom (SP)

(38) Department of Cellular Pathology, St George's University Hospitals NHS Foundation Trust St George's Hospital, London, United Kingdom (LB)

(39) Department of Neuropathology, Institute of Pathology, University Hospital Heidelberg, Heidelberg, Germany (TL, PS, DS, FS, AvD)

(40) Clinical Cooperation Unit Neuropathology, German Consortium for Translational Cancer Research (DKTK), German Cancer Research Center (DKFZ), Heidelberg, Germany (TL, PS, DS, FS, AvD)

* These authors share senior authorship

**Correspondence:** Emilie Le Rhun, MD, PhD, Department of Medical Oncology and Hematology, University Hospital and University of Zurich, Rämistrasse 100, CH-8091 Zürich, Switzerland, Phone +41 44 255 5500, E-mail: [emilie.lerhun@usz.ch](mailto:emilie.lerhun@usz.ch)

| Table S1. | H3 G34-mutant diffuse hemispheric glioma: patient and tumor characteristics by type of diagnostic method. |
| --- | --- |
| Table S2. | Adapted Visually AcceSAble Rembrandt Images (VASARI) criteria for the central imaging review (https://radiopaedia.org/articles/vasari-mri-feature-set). |
| Table S3. | Central imaging review of baseline MRI: H3 G34-mutant diffuse hemispheric glioma versus glioblastoma, IDH wildtype. |
| Table S4. | Main findings of the central pathology review (n=89). |
| Table S5. | Association of neuroimaging and neuropathology features by central review in 32 patients with diffuse hemispheric gliomas, H3 G34-mutant. |
| Table S6. | Copy number variations in diffuse hemispheric glioma, HR G34-mutant and selected other high-grade diffuse glioma types as detected by DNA methylation array-based copy number profiling: n (%). |
| Table S7. | Focal copy number underrepresentation affecting the *MGMT* locus as detect by EPIC array-based DNA copy number profiling: diffuse hemispheric glioma, HR G34-mutant, versus glioblastoma, IDH-wildtype. |
| Table S8. | Diffuse hemispheric glioma, H3 G34-mutant: details on treatment and outcome. |
| Table S9 | H3 G34-mutant diffuse hemispheric glioma: patient and tumor characteristics by age. |
| Table S10. | H3 G34-mutant diffuse hemispheric glioma: disease characteristics associated with long-term survival. |
| Table S11. | Univariate and multivariate analysis of prognostic factors for progression in patients with diffuse hemispheric glioma, H3 G34 mutant (Cox regression). |
| Table S12. | Univariate and multivariate analysis of prognostic factors for death in patients with diffuse hemispheric glioma, H3 G34 mutant – diagnosis confirmed with methylation and sequencing data (n=72) (Cox regression). |
| Table S13. | Univariate analysis of imaging markers for prognosis with regards to death in patients with diffuse hemispheric glioma, H3 G34-mutant (Cox regression). |
| Table S14. | Univariate analysis of type of H3 G34 mutation and histopathological markers for prognosis with regards to death in patients with diffuse hemispheric glioma, H3 G34-mutant (Cox regression). |
| Table S15. | Univariate analysis of molecular markers for prognosis with regards to death in patients with diffuse hemispheric glioma, H3 G34-mutant (Cox regression). |
| Table S16. | Patient characteristics stratified by *MGMT* promoter methylation and focal copy number underrepresentation affecting the *MGMT* locus (cut-off 0.5). |
| Table S17. | Patient characteristics stratified by *MGMT* promoter methylation and focal copy number underrepresentation affecting the *MGMT* locus (cut-off 0.75). |
| Table S18. | Prognostic associations of focal underrepresentation affecting the *MGMT* locus. |
| Figure S1. | Identification of patients with H3 G34-mutant diffuse hemispheric gliomas locally at the participating centers. |
| Figure S2. | Illustration of histological features in H3 G34-mutant diffuse hemispheric gliomas. |
| Figure S3. | tSNE plot and CNV profiles: H3 G34R- versus H3 G34V-mutant diffuse hemispheric gliomas. A: Annotations of the reference cohorts. B: tSNE plot and CNV profiles: H3 G34R- vs H3 G34V-mutant diffuse hemispheric gliomas. |
| Figure S4. | tSNE plot and CNV profiles: tumors of female versus male patients with diffuse hemispheric gliomas, H3 G34-mutant. |
| Figure S5. | tSNE plot and CNV profiles: age associations in patients with diffuse hemispheric gliomas, H3 G34-mutant. |
| Figure S6. | tSNE plot and CNV profiles: associations with tumor location and extension in patients with diffuse hemispheric gliomas, H3 G34-mutant. |
| Figure S7. | tSNE plot and CNV profiles: associations with contrast enhancement on central MRI review in patients with diffuse hemispheric gliomas, H3 G34-mutant. |
| Figure S8. | tSNE plot and CNV profiles: associations with diffusion restriction on central MRI review in patients with diffuse hemispheric gliomas, H3 G34-mutant. |
| Figure S9. | tSNE plot and CNV profiles: associations with necrosis on central MRI review in patients with diffuse hemispheric gliomas, H3 G34-mutant. |
| Figure S10. | tSNE plot and CNV profiles: associations with astrocytic versus PNET-like histology by central pathology review in patients with diffuse hemispheric gliomas, H3 G34-mutant. |
| Figure S11. | tSNE plot and CNV profiles: associations with histological detection of necrosis by central pathology review in patients with diffuse hemispheric gliomas, H3 G34-mutant. |
| Figure S12. | Diffuse hemispheric glioma, H3 G34-mutant: *MGMT* promoter methylation and focal copy number underrepresentation affecting the *MGMT* locus. |
| Figure S13. | DNA variants detected by gene panel sequencing: H3 G34R- versus H3 G34V-mutant diffuse hemispheric gliomas. |
| Figure S14. | *TP53* mutations in diffuse hemispheric glioma, H3 G34-mutant versus other gliomas: schematic p53 protein structure and variant location. |
| Figure S15. | tSNE plot and CNV profiles: associations with long term survivors in patients with diffuse hemispheric gliomas, H3 G34-mutant. |
| Figure S16. | tSNE plot and CNV profiles: associations with survival of patients with diffuse hemispheric gliomas, H3 G34-mutant. |
| Figure S17. | tSNE plot and CNV profiles: associations with survival restricted to deceased patients with diffuse hemispheric gliomas, H3 G34-mutant. |
| Note S1. | Detailed methods. |
| Note S2. | Reference cohorts. |
| Note S3. | Associations of histological differentiation with *PDGFR* or *CCND2* amplification. |

Table S1. **H3 G34-mutant diffuse hemispheric glioma: patient and tumor characteristics by type of diagnostic method.**

|  | **Patients diagnosed based on methylation profiling only**  **n=13** | **Patients with H3 G34R/V/M mutation confirmed by sequencing**  **n=101** | **P value** |
| --- | --- | --- | --- |
| **Age at first surgery** |  |  |  |
| Median (years) | 21 | 22 | 0.614 |
| Interquartile interval (Q1-Q3) | 17-25 | 18-31 |  |
| Range (minimum-maximum) | 12-45 | 8-70 |  |
| **Sex, n (%)** |  |  |  |
| Male | 9 (69) | 67 (66) | 0.835 |
| Female | 4 (31) | 34 (34) |  |
| **Age, n (%)** |  |  |  |
| < 18 years | 4 (31) | 22 (22) | 0.837 |
| 18-49 years | 9 (69) | 76 (75) |  |
| 50-59 years | 0 (0) | 2 (2) |  |
| 60-69 years | 0 (0) | 0 (0) |  |
| ≥ 70 years | 0 | 1 |  |
| **KPS at diagnosis, n (%)** |  |  |  |
| 90-100% | 6 (75) | 40 (50) | 0.301 |
| 70-80% | 2 (25) | 26 (33) |  |
| <70% | 0 (0) | 14 (18) |  |
| No data | 5 | 21 |  |
| **Extent of resection, n (%)** |  |  |  |
| Gross total resection | 4 (40) | 34 (35) | 0.201 |
| Incomplete | 5 (50) | 28 (29) |  |
| Biopsy | 1 (10) | 35 (36) |  |
| No data | 3 | 4 |  |
| ***MGMT* promoter status, n (%)** |  |  |  |
| Methylated | 9 (75) | 70 (75) | 0.984 |
| Unmethylated | 3 (25) | 23 (25) |  |
| No data | 1 | 8 |  |
| **Focal copy number under-representation affecting the *MGMT* locus, cut-off 0.75, n (%)** |  |  |  |
| Yes | 2 (17) | 9 (13) | 0.721 |
| No | 10 (83) | 61 (87) |  |
| No data | 1 | 31 |  |
| **First-line treatment – after surgery, n (%)** |  |  |  |
| No therapy | 1 (8) | 6 (6) | 0.858^a^ |
| Any therapy | 12 (92) | 88 (94) |  |
| Radiotherapy alone | 2 (15) | 1 (1) | 0.190^b^ |
| Temozolomide alone | 0 (0) | 3 (3) |  |
| Temozolomide/radiotherapy | 1 (8) | 6 (6) |  |
| Temozolomide/radiotherapy, followed by  Temozolomide | 9 (69) | 61 (65) |  |
| Temozolomide/radiotherapy, followed by  temozolomide-based regimen | 0 (0) | 13 (14)^c^ |  |
| Radiotherapy followed by temozolomide | 0 (0) | 1 (1) |  |
| Other | 0 (0) | 2 (2)^d^ |  |
| No data | 0 | 7 |  |
| **Outcome** |  |  |  |
| Median follow-up of surviving patients  (months, 95% CI, interquartile interval (Q1-Q3)) | 6.6  (0.0-19.9, 0.5-14.1) | 14.9  (8.7-21.2, 8.6-31.7) | 0.115 |
|  |  |  |  |
| Events (progression) | 9 | 70 |  |
| No progression during follow-up, n | 0 | 27 |  |
| No data on progression-free survival, n | 4 | 2 |  |
| Median progression-free survival  (months, 95% CI, interquartile interval (Q1-Q3)) | 8.2  (6.1-10.3, 6.6-25.1) | 9.7  (7.1-12.4, 6.3-17.7) | 0.872 |
|  |  |  |  |
| Events (death) | 9 | 62 |  |
| No data on overall survival, n | 0 | 2 |  |
| Alive or lost to follow-up at time of the analysis, n | 4 | 37 |  |
| Median overall survival (months, 95% CI, interquartile interval (Q1-Q3)) | 26.0  (0.0-58.0, 14.3-61.4) | 19.4  (13.9-25.0, 12.8-39.4) | 0.502 |

^a^, between no therapy and any therapy;

^b^, between no therapy and the different treatment options;

^c^, temozolomide/radiotherapy→temozolomide plus lomustine (CeTeG) (n=6); temozolomide/radiotherapy-based study (n=7);

^d^, carboplatin plus VP16, then radiotherapy then temozolomide (initial diagnosis of medulloblastoma) (n=1), temozolomide/radiotherapy→unknown (n=1);

Table S2. **Adapted Visually AcceSAble Rembrandt Images (VASARI) criteria for the central imaging review** (<https://radiopaedia.org/articles/vasari-mri-feature-set>)

| ITEMS | ADAPTED VASARI GUIDELINES * | ADAPTED VASARI GRADING FOR THIS STUDY |
| --- | --- | --- |
| Tumor location | Location of lesion.  geographic epicenter; the largest component of the tumor (either CET or nCET) (select one only). | Frontal  Temporal  Insular  Parietal  Occipital  Brainstem  Corpus callosum |
| Side of lesion center | Side of lesion epicenter. | Right  Left  Midline  Brainstem  Bilateral |
| Tumor extension | Number of lobe(s) involved. | 1 lobe / region  2 lobes / regions  3 lobes / regions  4 lobes / regions |
| Multifocality | Not anatomically connected and no connecting edema between lesion (2 mm normal brain or more in-between). | No  Yes |
| Edema | Visually, when scanning through the entire tumor volume, what proportion of the entire abnormality is estimated to represent vasogenic edema?: none, <5%, 6-33%, 34-67%, >67%  Edema should be greater in signal than nCET and somewhat lower in signal than CSF. Pseudopods are characteristic of edema. | None  <5%  6-33%  34-67%  >67% |
| Mass effect | Classification by Nestler et al., 2015 ^1^: “Volume effects of the tumor were measured by midline shift at the level of the third ventricle and using the following semiquantitative scale for ventricular wall impact.” | No compression  Wall compressed, CSF remains visible in the ventricular lumen  Opposite ventricular walls touch |
| Satellites | A satellite lesion is an area of enhancement within the region of signal abnormality surrounding the dominant lesion but not contiguous in any part with the major tumor mass. | No  Yes |
| Pial invasion | Enhancement of the overlying pia in continuity with enhancing or non-enhancing tumor. | No  Yes |
| Ependymal extension | Invasion of any adjacent ependymal surface in continuity with enhancing or non-enhancing tumor matrix. | No  Yes |
| Cortical involvement | Non-enhancing or enhancing tumor extending to the cortical mantle, or cortex is no longer distinguishable relative to subjacent tumor. | No  Yes |
| Deep white matter invasion | Enhancing or nCET tumor extending into the internal capsule, corpus callosum or brainstem. | No  Yes |
| Enhancement quality | Qualitative degree of contrast enhancement is defined as having all or portions of the tumor that demonstrate significantly higher signal on the postcontrast T1W images compared to precontrast T1W images. Mild/minimal = when barely discernable degree of enhancement is present relative to pre-contrast images. Marked/avid = obvious tissue enhancement. | None  Minimal/Mild  Marked/Avid |
| Relationship to neural stem cell regions | Classification by Lim et al. 2007 ^2^: contrast enhancement   1. in the cortex and subependymal region; 2. in the subependymal region, but not in the cortex;   III. in the cortex, but not in the subependymal region | I  II  III |
| CET crosses midline | Enhancing tissue crosses into contralateral hemisphere through white matter commissures (exclusive of herniated ipsilateral tissue). | No  Yes |
| Proportion enhancing | Contrast-enhancing tumor including potential necrosis (T1 CE). | 2D and semi-automatic volumetry |
| nCET crosses midline | nCET crosses into contralateral hemisphere through white matter commissures (exclusive of herniated ipsilateral tissue). | No  Yes |
| proportion nCET | Non-contrast-enhancing tumor excluding potential necrosis | 2D and semi-automatic volumetry |
| T1/FLAIR ratio (Use T2 if FLAIR is not provided) | Expansive = size of pre-contrast T1 abnormality (exclusive of signal intensity) approximates size of FLAIR abnormality.  Mixed = Size of T1 abnormality moderately less than FLAIR envelope;  Infiltrative = Size of precontrast T1 abnormality much smaller than size of FLAIR abnormality. | Expansive  Mixed  Infiltrative |
| Diffusion characteristics | Predominantly facilitated or restricted diffusion in the enhancing or nCET portion of the tumor (Based on ADC map). [Rate CET alone when present, otherwise use nCET].  Indeterminate = unsure.  Mixed = relatively equal proportion of facilitated and restricted. | Facilitated  Restricted  Mixed |
| Necrosis | Visually, when scanning through the entire tumor volume, what proportion of the tumor is estimated to represent necrosis. Necrosis is defined as a region within the tumor that does not enhance or shows markedly diminished enhancement, is high on T2W and proton density images, is low on T1W images, and has an irregular border. Assuming that the entire abnormality may be comprised of: (1) an enhancing component, (2) a non-enhancing component, (3) a necrotic component and (4) a edema component. | Absence  Presence, if presence:  2D and semi-automatic volumetry |
| Size of the whole lesion |  | Overall lesion  2D and semi-automatic volumetry |
| Cysts | Cysts are well defined, rounded, often eccentric regions of very bright T2W signal and low T1W signal essentially matching CSF signal intensity, with very thin, regular, smooth, nonenhancing or regularly enhancing walls, possibly with thin, regular, internal septations.  Nestler et al., 2015 ^1^: “The presence of tumor cysts was assumed when mostly round or oval-shaped lesions with T2w hyperintense and T1w hypointense signals, together with a delineated rim, were observed. The occurrence and number of cysts were noted, and the size of the largest cyst was measured.”  Lesion must be 5 mm in at least one dimension or larger  2D and semi-automatic volumetry. | Absence  Presence, if presence:  2D and semi-automatic volumetry |
| Hemorrhage | Intrinsic hemorrhage in the tumor matrix. Any intrinsic foci of low signal on T2WI or high signal on T1WI. | T2, SWI  Hemorrhage:  2D and semi-automatic volumetry  SWI changes: 1. / 2. |
| Calvarial remodelling | Erosion of inner table of skull (possibly a secondary sign of slow growth) | None=0,  Yes=1; Erosion of inner table of skull (possibly a secondary sign of slow growth) |

*References are cited if VASARI guidelines were not used

Table S3. **Central imaging review of baseline MRI: H3 G34-mutant diffuse hemispheric glioma versus glioblastoma, IDH wildtype.**

|  | All patients with H3 G34-mutant tumors  n=40 | | Patients with H3 G34R-mutant tumors  n=32 | | Control cohort  n=50^a^ | P value  (All G34 versus control cohort) |
| --- | --- | --- | --- | --- | --- | --- |
| LOCATION | | | | |  |  |
| Tumor location (tumor epicenter): n (%) | | | | |  |  |
| Frontal | 11 (28) | | 8 (25) | | 16 (32) | 0.239 |
| Temporal | 12 (30) | | 8 (25) | | 23 (46) |  |
| Insular | 3 (8) | | 2 (6) | | 2 (4) |  |
| Parietal | 10 (25) | | 10 (31) | | 6 (12) |  |
| Occipital | 2 (5) | | 2 (6) | | 0 (0) |  |
| Brain stem | 0 (0) | | 0 (0) | | 0 (0) |  |
| Corpus callosum | 2 (5) | | 2 (6) | | 3 (6) |  |
| Extent of tumor location: n (%)3 | | | | | |  |
| 1 lobe / region | 18 (45) | | 14 (44) | | 30 (60) | 0.507 |
| 2 lobes / regions | 13 (32) | | 10 (31) | | 13 (26) |  |
| 3 lobes / regions | 7 (18) | | 6 (19) | | 6 (12) |  |
| 4 lobes / regions | 2 (5) | | 2 (6) | | 1 (2) |  |
| Side of lesion center: n (%) | | | | | |  |
| Right | 17 (43) | | 15 (47) | | 20 (40) | 0.085 |
| Left | 21 (53) | | 15 (47) | | 21 (42) |  |
| Midline | 1 (3) | | 1 (3) | | 0 (0) |  |
| Bilateral | 1 (3) | | 1 (3) | | 9 (18) |  |
| Multifocality: n (%) defined as not anatomically connected and no connecting tumoral-related changes (either enhancing or non-enhancing) between lesion (2 mm normal brain or more in-between) | | | | | | |
| No | 36 (92) | | 28 (90) | | 40 (80) | 0.103 |
| Yes | 3 (8) | | 3 (10) | | 10 (20) |  |
| Not assessable | 1 (-) | | 1 (-) | | 0 (-) |  |
| TUMOR CHARACTERISTICS | | | | | | |
| Edema: n (%)  defined visually, when scanning through the entire tumor volume, by the proportion of the entire abnormality estimated to represent vasogenic edema | | | | | | |
| None | 5 (13) | | 5 (16) | | 0 (0) | <0.001 |
| <5% | 12 (30) | | 10 (31) | | 7 (14) |  |
| 6-33% | 16 (40) | | 12 (38) | | 17 (34) |  |
| 34-67% | 7 (18) | | 5 (16) | | 16 (32) |  |
| >67% | 0 (0) | | 0 (0) | | 10 (20) |  |
| Mass effect: n (%) | | | | | |  |
| No compression of ventricular walls | 4 (10) | | 4 (13) | | 6 (12) | 0.284 |
| Ventricular wall compressed, CSF remains visible in the ventricular lumen | 30 (75) | | 24 (75) | | 30 (60) |  |
| Opposite ventricular walls touch | 6 (15) | | 4 (13) | | 14 (28) |  |
| Satellites: n (%) | | | | | |  |
| No | 32 (82) | | 25 (81) | | 31 (62) | 0.039 |
| Yes | 7 (18) | | 6 (19) | | 19 (38) |  |
| Not assessable | 1 (-) | | 1 (-) | | 0 |  |
| Pial invasion: n (%) | | | | | |  |
| No | 9 (23) | | 6 (19) | | 19 (38) | 0.133 |
| Yes | 30 (77) | | 25 (81) | | 31 (62) |  |
| Not assessable | 1 (-) | | 1 (-) | | 0 |  |
| Ependymal extension: n (%) | | | | | |  |
| No | 19 (48) | | 13 (42) | | 20 (40) | 0.476 |
| Yes | 21 (53) | | 19 (59) | | 30 (60) |  |
| Cortical involvement: n (%) | | | | |  |  |
| No | 4 (10) | | 3 (10) | | 8 (16) | 0.405 |
| Yes | 36 (90) | | 29 (91) | | 42 (84) |  |
| Deep white matter invasion: n (%) | | | | | |  |
| No | 16 (40) | | 13 (41) | | 31 (62) | 0.038 |
| Yes | 24 (60) | | 19 (59) | | 19 (38) |  |
| Size of the whole lesion: median (interquartile interval (Q1-Q3)) | | | | | | |
| D1xD2 in cm^2^  Number of MRI assessed: n | 30 (21-45)  40 | | 27 (17-45)  32 | | 42 (29-59)  50 | 0.013 |
| D1xD2xD3 in cm^3^  Number of MRI assessed: n  Not assessable (n) | 163 (88-245)  39  1 | | 154 (78-244)  31  1 | | 236 (143-388)  50  - | 0.014 |
| Contrast enhancement | | | | | |  |
| Enhancement quality: n (%) |  | |  | |  |  |
| None | 7 (19) | | 6 (21) | | 0 (0) | <0.001 |
| Minimal/Mild | 17 (47) | | 13 (45) | | 10 (20) |  |
| Marked/Avid | 12 (33) | | 10 (35) | | 40 (80) |  |
| Not assessable | 4 (-) | | 3 (-) | | 0 (-) |  |
|  |  | |  | |  |  |
| Contrast enhancement dimensions: median (interquartile interval (Q1-Q3)) | | | | | | |
| *D1xD2 in cm^2^*  Number of MRI assessed: n | 8 (1-16)  29 | | 7 (1-17)  23 | | 21 (14-32)  50 | <0.001 |
| Not assessable: n | 4 | | 3 | | - |  |
| Not applicable (no contrast enhancement): n | 7 | | 6 | | - |  |
| *D1xD2xD3 in cm^3^*  Number of MRI assessed: n | 22 (2-52)  29 | | 21 (2-50)  23 | | 78 (46-145)  50 | <0.001 |
| Not assessable: n | 4 | | 3 | | - |  |
| Not applicable (no contrast enhancement): n | 7 | | 6 | | - |  |
|  |  | |  | |  |  |
| Contrast enhancement relationship to neural stem cell regions: n (%) | | | | |  |  |
| In the cortex and subependymal region | 8 (29) | | 7 (30) | | 20 (43) | 0.278 |
| In the subependymal region, but not in the cortex | 8 (29) | | 6 (26) | | 7 (15) |  |
| In the cortex, but not in the subependymal region | 12 (43) | | 9 (44) | | 20 (43) |  |
| Not assessable | 5 (-) | | 0 (-) | | 3 (-) |  |
| Not applicable (no contrast enhancement) | 7 (-) | | 9 (-) | | 0 (-) |  |
|  |  | |  | |  |  |
| Contrast enhancement crossing the midline: n (%) | | | | |  |  |
| No | 25 (86) | | 20 (87) | | 39 (78) | 0.370 |
| Yes | 4 (14) | | 3 (13) | | 11 (22) |  |
| Not assessable | 4 (-) | | 0 (-) | | 0 (-) |  |
| Not applicable (no contrast enhancement) | 7 (-) | | 9 (-) | | 0 (-) |  |
| Non-contrast-enhanced tumor | | | | | |  |
| Non-contrast-enhanced tumor dimensions: median (interquartile interval (Q1-Q3)) | | | | | |  |
| *D1xD2 in cm^2^*  Number of MRI assessed: n | 15 (6-28)  29 | | 15 (5-31)  24 | | 20 (5-34)  16 | 0.635 |
| Not assessable: n | 11 | | 8 | | 34 |  |
|  |  | |  | |  |  |
| *D1xD2xD3 in cm^3^*  Number of MRI assessed: n | 39 (11-112)  29 | | 38 (10-132)  24 | | 80 (7-152)  16 | 0.602 |
| Not assessable: n | 11 | | 8 | | 34 |  |
|  |  | |  | |  |  |
| Non-contrast-enhanced tumor relationship to neural stem cell regions: n (%) | | | | |  |  |
| In the cortex and subependymal region | 15 (48) | | 14 (54) | | 5 (33) | 0.362 |
| In the subependymal region, but not in the cortex | 4 (13) | | 3 (12) | | 5 (33) |  |
| In the cortex, but not in the subependymal region | 11 (36) | | 8 (31) | | 4 (27) |  |
| Neither cortex nor ependymal involvement | 1 (3) | | 1 (4) | | 1 (7) |  |
| Not assessable | 9 (-) | | 6 (-) | | 35 (-) |  |
|  |  | |  | |  |  |
| Non-contrast-enhanced tumor crosses midline: n (%) | | | | |  |  |
| No | 23 (74) | | 18 (72) | | 8 (50) | 0.097 |
| Yes | 8 (26) | | 7 (28) | | 8 (50) |  |
| Not assessable | 9 (-) | | 7 (-) | | 34 (-) |  |
| T1/FLAIR ratio: n (%)  defined as size of pre-contrast T1 abnormality (exclusive of signal intensity) versus size of FLAIR abnormality | | | | | | |
| Expansive | 22 (67) | 16 (64) | | 43 (86) | | 0.106 |
| Mixed | 7 (21) | 6 (24) | | 5 (10) | |  |
| Infiltrative | 4 (12) | 3 (12) | | 2 (4) | |  |
| Not assessable | 7 (-) | 7 (-) | | 0 (-) | |  |
| Diffusion: n (%) |  |  | |  | |  |
| Facilitated | 4 (13) | 3 (13) | | 4 (8) | | 0.287 |
| Restricted | 14 (47) | 11 (46) | | 17 (34) | |  |
| Mixed | 12 (40) | 10 (42) | | 29 (58) | |  |
| Not assessable | 10 (-) | 8 (-) | | | 0 (-) |  |
| Necrosis | | | | |  |  |
| No: n (%) | 22 (56) | | 18 (58) | | 5 (10) | <0.001 |
| Yes: n (%) | 17 (44) | | 13 (42) | | 44 (90) |  |
| Not assessable: n (%) | 1 (-) | | 1 (-) | | 1 (-) |  |
|  |  | |  | |  |  |
| Necrosis: median (interquartile interval (Q1-Q3)) | | |  | |  |  |
| *D1xD2 in cm^2^*  Number MRI assessed: n | 2 (0-8)  17 | | 2 (0.3-8)  13 | | 5 (2-9)  44 | 0.108 |
| Not assessable: n | 23 | | 18 | | 6 |  |
|  |  | |  | |  |  |
| *D1xD2xD3 in cm^3^*  Number of MRI assessed: n | 4 (0-12)  16 | | 4 (0.4-12)  12 | | 9 (2-25)  44 | 0.070 |
| Not assessable: n | 24 | | 19 | | 6 |  |
|  |  | |  | |  |  |
| Ratio volume necrosis/volume tumor (%)  Number of MRI assessed: n | 1.2 (0-9)  16 | | 1 (0-11)  12 | | 3.3 (1-10)  44 | 0.135 |
| Not assessable: n | 24 | | 20 | | 6 |  |
|  |  | |  | |  |  |
| Ratio volume necrosis/contrast enhanced  tumor (%)  Number of MRI assessed: n | 11 (5-28)  16 | | 14 (5-28)  12 | | 7 (4-28)  44 | 0.658 |
| Not assessable: n | 24 | | 20 | | 6 |  |
| Cyst: n (%) |  | |  | |  |  |
| Absent | 31 (78) | | 25 (78) | | 43 (86) | 0.295 |
| Present | 9 (23) | | 7 (22) | | 7 (14) |  |
| 1 cyst | 7 (-) | | 5 (-) | | 6 (-) |  |
| 2 cysts | 2 (-) | | 2 (-) | | 1 (-) |  |
|  |  | |  | |  |  |
| Largest cyst: median (interquartile interval (Q1-Q3)) | | |  | |  |  |
| Largest cyst D1xD2 cm^2^  Number of MRI assessed: n | 3 (1-12)  9 | | 5 (2-13)  7 | | 2 (1-4)  7 | 0.299 |
| Not applicable (no cyst): n | 31 | | 24 | | 43 |  |
| Largest cyst D1xD2xD3 cm^3^  Number of MRI assessed: n | 9 (2-41)  9 | | 11 (4-45)  7 | | 3 (1-8)  7 | 0.091 |
| Not applicable (no cyst): n | 31 | | 24 | | 43 |  |
| Hemorrhage: n (%) | | | | | |  |
| No | 27 (69) | | 22 (69) | | 28 (56) | 0.202 |
| Yes | 12 (31) | | 10 (31) | | 22 (44) |  |
| not assessable | 1 (-) | | 0 (-) | | 0 (-) |  |
|  |  | |  | |  |  |
| Hemorrhage median (interquartile interval (Q1-Q3)) | | |  | |  |  |
| *D1xD2 cm^2^*  Number of MRI assessed: n | 7 (3-16)  12 | | 7 (2-15)  10 | | 1 (1-3)  22 | 0.003 |
| Not assessable: n | 28 | | 19 | | 28 |  |
|  |  | |  | |  |  |
| *D1xD2xD3 cm^3^*  Number of MRI assessed: n | 19 (5-61)  12 | | 20 (3-51)  10 | | 1 (1-4)  22 | 0.002 |
| Not assessable: n | 28 | | 21 | | 21 |  |
| Calvarial remodeling: n (%)  defined as an erosion of inner table of skull | | |  | |  |  |
| None | 32 (80) | | 26 (81) | | 50 (100) | 0.004 |
| Yes | 7 (18) | | 5 (16) | | 0 |  |
| Suspicious | 1 (3) | | 1 (3) | | 0 |  |

^a^, The control cohort consisted of patients with glioblastoma, IDH wildtype, median age 60.5 years, 38 males (76%), median survival 12.4 months (95% CI 11.0-13.8, interquartile interval (Q1-Q3) 7.7-18.2).

Table S4. **Main findings of the central pathology review (n=89) of diagnostic samples.**

|  | **All**  **n=89** | **Confirmed H3 G34R mutation only**  **n=75** |
| --- | --- | --- |
| **n (%)** |  |  |
| FFPE | 51 (57) | 36 (48) |
| e-slides | 38 (43) | 39 (52) |
| **Cell density** |  |  |
| Low | 2 (2) | 2 (3) |
| Moderate | 26 (29) | 22 (29) |
| High | 61 (69) | 51 (68) |
| **Pleomorphism** |  |  |
| Low | 7 (8) | 6 (8) |
| Moderate | 69 (78) | 58 (77) |
| High | 13 (15) | 11 (15) |
| **Differentiation** |  |  |
| Astrocytic | 61 (69) | 51 (68) |
| Astrocytic with PNET-like component and PNET-like | 28 (31) | 24 (32) |
| **Mitoses (x/10 high power fields)** |  |  |
| No | 6 (8) | 4 (6) |
| 1-9 | 30 (41) | 7 (43) |
| 10 or more | 38 (51) | 31 (50) |
| Not assessable | 15 (-) | 13 (-) |
| **Necrosis** |  |  |
| Yes | 27 (30) | 23 (31) |
| Yes, serpentine | 13 (15) | 9 (12) |
| No | 49 (55) | 43 (57) |
| **Microvascular proliferation** |  |  |
| Yes | 53 (60) | 44 (59) |
| No | 36 (40) | 31 (41) |
| **Necrosis and/or microvascular proliferation** |  |  |
| No | 30 (34) | 27 (36) |
| Yes | 59 (66) | 48 (64) |
| **Multinuclear giant cells** |  |  |
| Yes, single | 21 (24) | 19 (25) |
| Yes, prominent | 10 (11) | 8 (11) |
| No | 58 (65) | 48 (64) |
| **Focal calcification** |  |  |
| Yes | 7 (8) | 5 (7) |
| No | 82 (92) | 70 (93) |

The 14 patients with central review, but without documented G34R mutation included patients with the G34V mutation (n=8), the G34M mutation (n=1), lack of data on type of mutation (n=4) and absence of mutation (n=1). FFPE, formalin-fixed and paraffin-embedded tissue sections; PNET-like, primitive neuroectodermal tumor-like.

Table S5. **Association of neuroimaging and neuropathology features by central review in 32 patients with diffuse hemispheric gliomas, H3 G34-mutant (n, %).**

| **MRI**  **Histology** | **No necrosis on MRI** | **Necrosis on MRI** | **Necrosis on MRI not assessable** | **Total** |
| --- | --- | --- | --- | --- |
|  |  |  |  |  |
| **No necrosis on histology** | 14 (78%) | 7 (54%) | 1 | 22 |
| **Necrosis on histology** | 4 (22%) | 6 (46%) | 0 | 10 |
| P value (Chi square) | 0.160 |  |  |  |
|  |  |  |  |  |
| **No microvascular proliferation** | 10 (57%) | 4 (31%) | 0 | 14 |
| **Microvascular proliferation** | 8 (44%) | 9 (69%) | 1 | 18 |
| P value (Chi square) | 0.171 |  |  |  |
|  |  |  |  |  |
| **Neither necrosis nor microvascular proliferation** | 9 (50%) | 3 (23%) | 0 | 12 |
| **Necrosis and/or microvascular proliferation** | 9 (50%) | 10 (77%) | 1 | 20 |
| P value (Chi square) | 0.129 |  |  |  |

| **MRI**  **Histology** | **No contrast enhancement on MRI** | **Mild contrast enhancement on MRI** | **Avid contrast enhancement on MRI** | **Contrast enhancement on MRI not assessable** | **Total** |
| --- | --- | --- | --- | --- | --- |
|  |  |  |  |  |  |
| **No necrosis on histology** | 7 (100%) | 8 (67%) | 5 (50%) | 2 | 22 |
| **Necrosis on histology** | 0 | 4 (33%) | 5 (50%) | 1 | 10 |
| P value (Chi square) | 0.088 |  |  |  |  |
|  |  |  |  |  |  |
| **No microvascular proliferation** | 5 (71%) | 7 (58%) | 2 (20%) | 0 | 14 |
| **Microvascular proliferation** | 2 (29%) | 5 (42%) | 8 (80%) | 3 | 18 |
| P value (Chi square) | 0.075 |  |  |  |  |
|  |  |  |  |  |  |
| **Neither necrosis nor microvascular proliferation** | 5 (71%) | 5 (42%) | 2 (20%) | 0 | 12 |
| **Necrosis and/or microvascular proliferation** | 2 (29%) | 7 (58%) | 8 (80%) | 3 | 20 |
| P value (Chi square) | 0.106 |  |  |  |  |

Table S6**. Copy number variations in diffuse hemispheric glioma, H3 G34-mutant and selected other high-grade diffuse glioma types (reference cohort 3) as detected by DNA methylation array-based copy number profiling: n (%).**

| **Gene** | Methylation class diffuse hemispheric glioma, **H3 G34-mutant** (n=82) | | | Methylation class **glioblastoma**, IDH wildtype, subclass **mesenchymal** (n=20) | | | Methylation class **glioblastoma**, IDH wildtype, subclass **RTK1** (n=20) | | | Methylation class **glioblastoma**, IDH wildtype, subclass **RTK2** (n=20) | | | Methylation class IDH glioma, subclass 1p/19q codeleted **oligodendroglioma** (n=20) | | | Methylation class IDH glioma, subclass **astrocytoma** (n=20) | | | Methylation class IDH glioma, subclass **high grade astrocytoma** (n=20) | | |
| --- | --- | --- | --- | --- | --- | --- | --- | --- | --- | --- | --- | --- | --- | --- | --- | --- | --- | --- | --- | --- | --- |
|  | HOM DEL* |  | AMP | HOM DEL |  | AMP | HOM DEL |  | AMP | HOM DEL |  | AMP | HOM DEL |  | AMP | HOM DEL |  | AMP | HOM DEL |  | AMP |
| *CCND1* | 0 |  | 1 (1) | 0 |  | 0 | 0 |  | 0 | 0 |  | 0 | 0 |  | 0 | 0 |  | 0 | 0 |  | 0 |
| *CCND2* | 0 |  | 6 (7) | 0 |  | 0 | 0 |  | 2 (10) | 0 |  | 0 | 0 |  | 0 | 0 |  | 0 | 0 |  | 0 |
| *CDK4* | 0 |  | 1 (1) | 0 |  | 1 (5) | 0 |  | 7 (35) | 0 |  | 3 (15) | 0 |  | 0 | 0 |  | 0 | 0 |  | 2 (10) |
| *CDK6* | 0 |  | 12 (15) | 0 |  | 0 | 0 |  | 1 (5) | 0 |  | 0 | 0 |  | 0 | 0 |  | 0 | 0 |  | 0 |
| *CDKN2A/B* | 17 (21) |  | 1 (1) | 8 (40) |  | 0 | 9 (45) |  | 0 | 18 (90) |  | 0 | 0 |  | 0 | 0 |  | 0 | 8 (40) |  | 0 |
| *EGFR* | 0 |  | 6 (7) | 0 |  | 8 | 0 |  | 1 (5) | 0 |  | 19 (95) | 0 |  | 0 | 0 |  | 0 | 0 |  | 0 |
| *GLI2* | 1 (1) |  | 0 | 0 |  | 0 | 0 |  | 0 | 0 |  | 0 | 0 |  | 0 | 0 |  | 0 | 0 |  | 0 |
| *MDM2* | 0 |  | 0 | 0 |  | 0 | 0 |  | 6 (30) | 0 |  | 0 | 0 |  | 0 | 0 |  | 0 | 0 |  | 0 |
| *MDM4* | 0 |  | 2 (2) | 0 |  | 0 | 0 |  | 1 (5) | 0 |  | 2 (10) | 0 |  | 0 | 0 |  | 0 | 0 |  | 0 |
| *MET* | 0 |  | 1 (1) | 0 |  | 0 | 0 |  | 3 | 0 |  | 0 | 0 |  | 0 | 0 |  | 0 | 1 (5) |  | 2 (10) |
| *MGMT*** | 21 (26) |  | 0 | 1 (5) |  | 0 | 0 |  | 0 | 1 (5) |  | 0 | 0 |  | 0 | 0 |  | 0 | 1 (5) |  | 0 |
| *MYC* | 0 |  | 2 (2) | 0 |  | 0 | 0 |  | 0 | 0 |  | 0 | 0 |  | 0 | 0 |  | 0 | 0 |  | 2 (10) |
| *MYCN* | 0 |  | 4 (5) | 0 |  | 0 | 0 |  | 0 | 0 |  | 0 | 0 |  | 0 | 0 |  | 0 | 0 |  | 1 (5) |
| *NF1* | 1 (1) |  | 0 | 0 |  | 0 | 1 (5) |  | 0 | 1 (5) |  | 0 | 0 |  | 0 | 0 |  | 0 | 0 |  | 0 |
| *PDGFRA* | 1 (1) |  | 16 (20) | 0 |  | 0 | 0 |  | 3 | 1 (5) |  | 2 (10) | 0 |  | 0 | 0 |  | 0 | 0 |  | 3 (15) |
| *PPM1D* | 1 (1) |  | 0 | 0 |  | 0 | 0 |  | 0 | 1 (5) |  | 0 | 0 |  | 0 | 0 |  | 0 | 0 |  | 0 |
| *PTCH1* | 0 |  | 1 (1) | 0 |  | 0 | 0 |  | 0 | 0 |  | 0 | 0 |  | 0 | 0 |  | 0 | 0 |  | 0 |
| *PTEN* | 3 (4) |  | 1 (1) | 0 |  | 0 | 0 |  | 0 | 3 |  | 0 | 0 |  | 0 | 0 |  | 0 | 0 |  | 0 |
| *RB1* | 18 (22) |  | 0 | 0 |  | 0 | 3 |  | 0 | 0 |  | 0 | 3 (15) |  | 0 | 1 (5) |  | 0 | 2 (10) |  | 0 |
| *SMARCB1* | 2 (2) |  | 0 | 0 |  | 0 | 0 |  | 0 | 0 |  | 0 | 0 |  | 0 | 0 |  | 0 | 0 |  | 0 |
| *TERT* | 2 (2) |  | 0 | 0 |  | 0 | 0 |  | 0 | 0 |  | 1 (5) | 0 |  | 0 | 0 |  | 0 | 1 (5) |  | 0 |
| *TP53* | 5 (6) |  | 0 | 0 |  | 0 | 0 |  | 0 | 0 |  | 0 | 0 |  | 0 | 0 |  | 0 | 0 |  | 0 |

*HOM DEL, homozygous deletion; AMP amplification, ** focal copy number underrepresentation affecting the *MGMT* locus.

Table S7. **Focal copy number underrepresentation affecting the *MGMT* locus as detect by EPIC array-based DNA copy number profiling: diffuse hemispheric glioma, HR G34-mutant, versus glioblastoma, IDH-wildtype, according to distinct copy number thresholds (n, %).**

|  | **Diffuse hemispheric glioma, H3 G34-mutant** | **MES**  **glioblastoma** | **RTK1**  **glioblastoma** | **RTK2**  **glioblastoma** |
| --- | --- | --- | --- | --- |
|  |  |  |  |  |
| This study | 82 | 20 | 20 | 20 |
| Treshold <-0.5 (log2) | 21 (25.6%) | 1 (5%) | 0 | 1 (5%) |
| Treshold <-0.75 (log2) | 11 (13.4%) | 0 | 0 | 1 (5%) |
|  |  |  |  |  |
| Reference Reuss et al. 2024 ^3^ |  | 2267 | 2267 | 3924 |
| Treshold <-0.5 (log2) | n.a. | 82 (3.6%) | 433 (19.1%) | 1065 (27.1%) |
| Treshold <-0.75 (log2) | n.a. | 7 (0.3%) | 84 (3.7%) | 229 (5.8%) |

n.a.: not applicable

Table S8. **Diffuse hemispheric glioma, H3 G34-mutant: details on treatment and outcome.**

|  | **Diffuse hemispheric glioma, H3 G34 mutant**  **n=114** |
| --- | --- |
| **First line treatment** |  |
| Completed as planned | 37 (32) |
| Prematurely stopped | 50 (44) |
| No first line treatment | 7 (6) |
| No data | 16 (14) |
| Treatment ongoing | 4 (4) |
| **Reason for premature stop of first line treatment** | **n=50** |
| Progression | 40 (80) |
| Toxicity | 5 (10) |
| Alteration of the general status | 5 (10) |
| **KPS at progression (1)** | **n=58** |
| Median | 80 |
| Range (interquartile interval (Q1-Q3)) | 20-100 (70-90) |
| **Treatment at progression^a^** | **n=82** |
| None | 10 (12) |
| Any treatment | 68 (83) |
| Surgery alone | 3 (4) |
| Radiotherapy alone | 2 (2) |
| Local treatment (surgery or radiotherapy, no data on systemic treatment) | 7 (10) |
| Systemic treatment alone | 30 (36) |
| Surgery plus radiotherapy | 2 (2) |
| Surgery plus systemic treatment | 12 (15) |
| Radiotherapy plus systemic treatment | 6 (7) |
| Surgery plus radiotherapy plus systemic treatment | 6 (7) |
| No data | 4 (5) |

^a^, 82 patients had a documented first progression, but 3 patients had no information on timepoint of progression and were omitted in Table 1. Data on KPS were available for 58 patients. CI, confidence interval; KPS, Karnofsky Performance Status. Data are provided in numbers and percentages in brackets.

Table S9. **H3 G34-mutant diffuse hemispheric glioma: patient and tumor characteristics by age.**

|  | **Age < 18 years**  **n=26** | **Age ≥ 18 years**  **n=88** | **P value** |
| --- | --- | --- | --- |
| **Age at first surgery** |  |  |  |
| Median (years) | 14 | 25 | < 0.001 |
| Interquartile interval (Q1-Q3) | 12-16 | 20-34 |  |
| Range (minimum-maximum) | 8-17 | 18-70 |  |
| **Sex, n (%)** |  |  |  |
| Male | 19 (73) | 57 (65) | 0.430 |
| Female | 7 (27) | 31 (35) |  |
| **KPS at diagnosis, n (%)** |  |  |  |
| 90-100% | 13 (72) | 33 (47) | 0.142 |
| 70-80% | 4 (22) | 24 (34) |  |
| <70% | 1 (6) | 13 (19) |  |
| No data | 8 | 18 |  |
| **Extent of resection, n (%)** |  |  |  |
| Gross total resection | 6 (26) | 32 (38) | 0.137 |
| Incomplete | 11 (48) | 22 (26) |  |
| Biopsy | 6 (26) | 30 (36) |  |
| No data | 3 | 4 |  |
| ***MGMT* promoter status, n (%)** |  |  |  |
| Methylated | 15 (68) | 64 (77) | 0.388 |
| Unmethylated | 7 (32) | 19 (23) |  |
| No data | 4 | 5 |  |
| **Focal copy number under-representation affecting the *MGMT* locus, cut-off 0.5, n (%)** |  |  |  |
| Yes | 6 (33) | 15 (23) | 0.395 |
| No | 12 (67) | 49 (77) |  |
| No data | 8 | 24 |  |
| **Focal copy number under-representation affecting the *MGMT* locus, cut-off 0.75, n (%)** |  |  |  |
| Yes | 4 (22) | 7 (11) | 0.215 |
| No | 14 (78) | 57 (89) |  |
| No data | 8 | 24 |  |
| **First-line treatment – after surgery, n (%)** |  |  |  |
| No therapy | 0 (0) | 7 (8) | 0.152^a^ |
| Any therapy | 23 (100) | 77 (92) |  |
| Radiotherapy alone | 1 (4) | 2 (2) | 0.752^b^ |
| Temozolomide alone | 0 (0) | 3 (4) |  |
| Temozolomide/radiotherapy | 1 (4) | 6 (7) |  |
| Temozolomide/radiotherapy, followed by  Temozolomide | 18 (78) | 52 (62) |  |
| Temozolomide/radiotherapy, followed by  temozolomide-based regimen | 3 (13)^c^ | 10 (12)^d^ |  |
| Radiotherapy followed by temozolomide | 0 (0) | 1 (1) |  |
| Other | 0 (0) | 2 (2)^e^ |  |
| No data | 3 | 4 |  |
| **Outcome** |  |  |  |
| Median follow-up of surviving patients  (months, 95% CI, interquartile interval (Q1-Q3)) | 14.1  (11.5-16.2, 6.9-18.2) | 14.1  (4.0-24.3, 8.2-31.7) | 0.932 |
|  |  |  |  |
| Events (progression) | 17 | 62 |  |
| No progression during follow-up, n | 1 | 3 |  |
| No data on progression-free survival, n | 8 | 23 |  |
| Median progression-free survival  (months, 95% CI, interquartile interval (Q1-Q3)) | 12.7  (9.5-18.9, 7.9-15.0) | 8.5  (7.1-9.9, 5.3-22.3) | 0.903 |
|  |  |  |  |
| Events (death) | 18 | 53 |  |
| No data on overall survival, n | 0 | 2 |  |
| Alive at last follow-up, nn | 8 | 33 |  |
| Median overall survival (months, 95% CI, interquartile interval (Q1-Q3)) | 17.3  (15.9-18.8, 14.3-26.0) | 24.1  (16.9-31.2, 13.6-54.7) | 0.209 |

^a^, between no therapy and any therapy;

^b^, between no therapy and the different treatment options;

^c^, temozolomide/radiotherapy→temozolomide plus lomustine (CeTeG) (n=1); temozolomide/radiotherapy-based study (n=2);

^d^, temozolomide/radiotherapy→temozolomide plus lomustine (CeTeG) (n=5); temozolomide/radiotherapy-based study (n=5);

^e^, carboplatin plus VP16, then radiotherapy then temozolomide (initial diagnosis of medulloblastoma) (n=1), temozolomide/radiotherapy→unknown (n=1);

Table S10. **H3 G34-mutant diffuse hemispheric glioma: disease characteristics associated with long-term survival.**

|  | **Patients with survival of 5 years or more**  **n=8** | **Patients deceased (known death) before 5 years**  **n=67** | **P value** |
| --- | --- | --- | --- |
| **Age at first surgery** |  |  |  |
| Median (years) | 29.5 | 20 | 0.030 |
| Interquartile interval (Q1-Q3) | 22-44 | 17-27 |  |
| Range (minimum-maximum) | 15-45 | 8-56 |  |
| **Sex, n (%)** |  |  |  |
| Male | 3 (38) | 52 (78) | 0.015 |
| Female | 5 (63) | 15 (22) |  |
| **Age, n (%)** |  |  |  |
| < 18 years | 1 (13) | 18 (27) |  |
| 18-49 years | 7 (88) | 47 (70) | 0.571 |
| 50-59 years | 0 | 2 (3) |  |
| 60-69 years | 0 | 0 |  |
| ≥ 70 years | 0 | 0 |  |
| **KPS at diagnosis, n (%)** |  |  |  |
| 90-100% | 5 (71) | 26 (51) | 0.546 |
| 70-80% | 1 (14) | 17 (33) |  |
| <70% | 1 (14) | 8 (16) |  |
| No data | 1 | 16 |  |
| **Extent of resection, n (%)** |  |  |  |
| Gross total resection | 5 (71) | 24 (37) | 0.120 |
| Incomplete | 2 (29) | 19 (29) |  |
| Biopsy | 0 | 22 (34) |  |
| Autopsy | 0 | 0 |  |
| No data | 1 | 2 |  |
| ***MGMT* promoter status, n (%)** |  |  |  |
| Methylated | 7 (88) | 40 (68) | 0.253 |
| Unmethylated | 1 (13) | 19 (32) |  |
| No data | 0 | 8 |  |
| **Focal copy number under-representation affecting the *MGMT* locus, cut-off 0.75, n (%)** |  |  |  |
| Yes | 2 (25) | 3 (7) | 0.102 |
| No | 6 (75) | 42 (93) |  |
| No data | 0 | 22 |  |
| **First-line treatment – after surgery, n (%)** |  |  |  |
| No therapy | 0 (0) | 4 (6) | 0.463^a^ |
| Any therapy | 8 (100) | 59 (94) |  |
| Radiotherapy alone | 0 | 3 (5) | 0.076^b^ |
| Temozolomide alone | 0 | 2 (3) |  |
| Temozolomide/radiotherapy | 0 | 6 (10) |  |
| Temozolomide/radiotherapy, followed by  temozolomide | 7 (88) | 40 (64) |  |
| Temozolomide/radiotherapy, followed by  temozolomide-based regimen | 0 | 8 (13)^c^ |  |
| Radiotherapy followed by temozolomide | 0 | 0 |  |
| Surgery alone with no planned further treatment | 1 (13) | 0 |  |
| No data | 0 | 4 |  |
| **Outcome** |  |  |  |
| Median follow-up of surviving patients  (months, 95% CI, interquartile interval (Q1-Q3) | 122.0  (80.6-163.4, 85.5-127.8) | -  - | - |
|  |  |  |  |
| Events (progression) | 8 | 51 |  |
| No progression during follow-up, n | 0 | 0 |  |
| No data on progression-free survival, n | 0 | 8 |  |
| Median progression-free survival  (months, 95% CI, interquartile interval (Q1-Q3)) | 46.8  (31.7-62.0, 7.4-62.1) | 9.4  (6.2-12.6, 5.3-15.0) | <0.001 |
|  |  |  |  |
| Events (death) | 4 | 67 |  |
| No data on overall survival, n | 0 | 0 |  |
| Alive or lost to follow-up at the time of the analysis, n | 4 | 0 |  |
| Median overall survival  (months, 95% CI, interquartile interval (Q1-Q3)) | 88.3  (undefined, 67.9-undefined) | 15.5  (13.4-17.6, 9.4-24.1) | <0.001 |

^a^, between no therapy and any therapy;

^b^, between no therapy and the different treatment options;

^c^, temozolomide/radiotherapy→temozolomide plus lomustine (CeTeG) (n=1); temozolomide/radiotherapy-based study (n=7);

Table S11. **Univariate and multivariate analysis of prognostic factors for progression in patients with diffuse hemispheric glioma, H3 G34** mutant (Cox regression).

|  | **Univariate analysis** | | | | | |  | | **Multivariate analysis** | | | |
| --- | --- | --- | --- | --- | --- | --- | --- | --- | --- | --- | --- | --- |
|  | **n (events)** | | **HR (95 % CI)** | | **P value** |  | | **n**  **(events)** | | **HR (95 % CI)** | | **P value** |
| **Age** |  | |  | |  |  | |  | |  | |  |
| < 18 years | 18 (17) | | 0.90 (0.60-1.78) | | 0.903 |  | | 9 (9) | | 0.88 (0.39-1.98) | | 0.751 |
| ≥ 18 years | 65 (62) | | 1 | | ref |  | | 48 (46) | | 1 | | ref |
| **Sex** |  | |  | |  |  | |  | |  | |  |
| Female | 27 (26) | | 0.53 (0.32-0.88) | | 0.013 |  | | 23 (22) | | 0.53 (0.27-1.03) | | 0.061 |
| Male | 56 (53) | | 1 | | ref |  | | 34 (33) | | 1 | | ref |
| **KPS** |  | |  | |  |  | |  | |  | |  |
| < 70% | 7 (7) | | 1.45 (0.64-3.30) | | 0.378 |  | | 5 (5) | | 1.37 (0.49-3.86) | | 0.549 |
| 70-80% | 23 (22) | | 1.11 (0.65-1.91) | | 0.698 |  | | 21 (20) | | 0.62 (0.32-1.19) | | 0.153 |
| 90-100% | 37 (35) | | 1 | | ref |  | | 31 (30) | | 1 | | ref |
| No data | 16 | | - | | - |  | | - | |  | |  |
| **Extent of resection** |  | |  | |  |  | |  | |  | |  |
| Gross total | 29 (29) | | 0.63 (0.34-1.16) | | 0.137 |  | | 23 (23) | | 0.34 (0.15-0.80) | | 0.013 |
| Incomplete | 27 (26) | | 0.99 (0.54-1.81) | | 0.970 |  | | 18 (18) | | 0.72 (0.32-1.58) | | 0.408 |
| Biopsy/Autopsy | 20 (18) | | 1 | | ref |  | | 16 (14) | | 1 | | ref |
| No data | 7 | | - | | - |  | | - | |  | |  |
| ***MGMT* promoter status** | | | |  | | |  | |  | |  |  |
| Methylated | 55 (51) | 0.44 (0.26-0.76) | | | 0.003 |  | | 39 (37) | | 0.43 (0.20-0.90) | | 0.026 |
| Unmethylated | 21 (21) | 1 | | | ref |  | | 18 (18) | | 1 | | ref |
| No data | 7 | - | | | - |  | | - | |  | |  |
| **First-line treatment** |  |  | | |  |  | |  | |  | |  |
| No therapy | 1 (1) | - | | | - |  | | 1 (1) | | - | | - |
| Any therapy | 82 (78) | - | | | - |  | | 56 (54) | | - | | - |

CI, confidence interval; HR, hazard ratio; KPS, Karnofsky performance status; MGMT, O^6^-methylguanine DNA methyltransferase; survival data are missing in 31 patients

Table S12. **Univariate and multivariate analysis of prognostic factors for death in patients with diffuse hemispheric glioma, H3 G34** mutant – diagnosis confirmed with methylation and sequencing data (n=72) (Cox regression).

|  | **Univariate analysis** | | | | |  | | **Multivariate analysis** | | | | | |  |
| --- | --- | --- | --- | --- | --- | --- | --- | --- | --- | --- | --- | --- | --- | --- |
|  | **n (events)** | | **HR (95 % CI)** | | **P value** | |  | | **n (events)** | | **HR (95 % CI)** | **P value** | | |
| **Age** |  | |  | |  | |  | |  | |  |  | | |
| < 18 years | 14 (9) | | 1.24 (0.59-2.61) | | 0.576 | |  | | 10 (6) | | 1.42 (0.54-3.72) | 0.475 | | |
| ≥ 18 years | 56 (33) | | 1 | | ref | |  | | 46 (29) | | 1 | ref | | |
| **Sex** |  | |  | |  | |  | |  | |  |  | | |
| Female | 33 (8) | | 0.32 (0.15-0.69) | | 0.004 | |  | | 21 (7) | | 0.31 (0.13-0.78) | 0.013 | | |
| Male | 47 (34) | | 1 | | ref | |  | | 35 (28) | | 1 | ref | | |
| **KPS** |  | |  | |  | |  | |  | |  |  | | |
| < 70% | 11 (7) | | 1.92 (0.90-4.08) | | 0.092 | |  | | 9 (7) | | 1.14 (0.31-4.18) | 0.842 | | |
| 70-80% | 16 (9) | | 1.20 (0.66-2.20) | | 0.552 | |  | | 16 (9) | | 0.82 (0.33-2.03) | 0.661 | | |
| 90-100% | 33 (20) | | 1 | | ref | |  | | 31 (19) | | 1 | ref | | |
| No data | 10 | | - | | - | |  | | - | |  |  | | |
| **Extent of resection** |  | |  | |  | |  | |  | |  |  | | |
| Gross total | 28 (19) | | 0.65 (0.30-1.41) | | 0.272 | |  | | 23 (16) | | 0.41 (0.14-1.17) | 0.096 | | |
| Incomplete | 21 (12) | | 0.82 (0.35-1.90) | | 0.640 | |  | | 18 (11) | | 0.36 (0.11-1.11) | 0.076 | | |
| Biopsy/Autopsy | 17 (10) | | 1 | | ref | |  | | 15 (8) | | 1 | ref | | |
| No data | 3 | | - | | - | |  | | - | |  |  | | |
| ***MGMT* promoter status** | | | |  | | |  | |  |  | |  |  |  |
| Methylated | 50 (26) | 0.53 (0.28-0.99) | | | 0.045 | |  | | 38 (20) | | 0.59 (0.24-1.43) | 0.244 |  |  |
| Unmethylated | 20 (16) | 1 | | | ref | |  | | 18 (15) | | 1 | ref |  |  |
| **First-line treatment** |  |  | | |  | |  | |  | |  |  |  |  |
| No therapy | 3 (3) | 1 | | | ref | |  | | 3 (3) | | 1 | ref |  |  |
| Any therapy | 65 (39) | 0.02 (0.00-0.12) | | | <0.01 | |  | | 53 (32) | | 0.04 (0.01-0.25) | < 0.001 |  |  |
| No data | 2 | - | | - | | |  | | - |  | |  |  |  |

CI, confidence interval; HR, hazard ratio; KPS, Karnofsky performance status; MGMT, O^6^-methylguanine DNA methyltransferase; survival data are missing in 2 patients

Table S13. **Univariate analysis of imaging markers for prognosis with regards to death in patients with diffuse hemispheric glioma, H3 G34-mutant (Cox regression) (n=112).^a^**

|  | **n (events)** | **HR (95% CI)** | **p-value** |
| --- | --- | --- | --- |
| **LOCATION** |  |  |  |
| **Tumor location (tumor epicenter)** |  |  |  |
| Frontal | 11 (6) | 1 | ref |
| Temporal | 12 (9) | 2.52 (0.82-7.70) | 0.105 |
| Insular | 3 (2) | - | - |
| Parietal | 9 (3) | 0.81 (0.20-3.26) | 0.761 |
| Occipital | 2 (2) | - | - |
| Brain stem | 0 (0) | - | - |
| Corpus callosum | 2 (0) | - | - |
| No data | 73 | - | - |
| **Extent of tumor location** |  |  |  |
| 1 lobe / region | 17 (11) | 1 | ref |
| 2 lobes / regions | 13 (6) | 1.20 (0.42-3.44) | 0.740 |
| 3 lobes / regions | 7 (3) | 0.91 (0.25-3.30) | 0.886 |
| 4 lobes / regions | 2 (2) | - | - |
| No data | 73 | - | - |
| **Side of lesion center** |  |  |  |
| Right | 16 (10) | 1.02 (0.44-2.38) | 0.957 |
| Left | 21 (12) | 1 | ref |
| Midline | 1 (0) | - | - |
| Brain stem | 0 (0) | - | - |
| Bilateral | 1 (0) | - | - |
| No data | 73 |  |  |
| **Multifocality***: defined as not anatomically connected and no connecting tumoral-related changes (either enhancing or non-enhancing) between lesion (2 mm normal brain or more in-between)* |  |  |  |
| No | 35 (21) | - | - |
| Yes | 3 (1) | - | - |
| No data | 74 | - | - |
| **TUMOR CHARACTERISTICS** |  |  |  |
| **Edema:** *defined visually, when scanning through the entire tumor volume, by the proportion of the entire abnormality estimated to represent vasogenic edema* |  |  |  |
| None | 5 (2) | 1 | ref |
| <5% | 11 (8) | 1.55 (0.33-7.40) | 0.580 |
| 6-33% | 16 (9) | 1.54 (0.33-7.27) | 0.587 |
| >33% | 7 (3) | 0.78 (0.12-4.92) | 0.789 |
| No data | 73 | - | - |
| **Mass effect** |  |  |  |
| No compression of ventricular walls | 4 (2) | - | - |
| Ventricular wall compressed, CSF remains visible in the ventricular lumen | 29 (16) | 1 | ref |
| Opposite ventricular walls touch | 6 (4) | 0.76 (0.25-2.29) | 0.622 |
| No data | 73 | - | - |
| **Satellites** |  |  |  |
| No | 31 (18) | 1 | ref |
| Yes | 7 (4) | 1.33 (0.44-4.03) | 0.610 |
| No data | 74 | - | - |
| **Pial invasion** |  |  |  |
| No | 9 (3) | 1 | ref |
| Yes | 29 (19) | 4.67 (1.04-21.01) | 0.044 |
| No data | 74 | - | - |
| **Ependymal extension** |  |  |  |
| No | 18 (11) | 1 | ref |
| Yes | 21 (11) | 1.12 (0.48-2.60) | 0.793 |
| No data | 73 | - | - |
| **Cortical involvement** |  |  |  |
| No | 4 (1) | - | - |
| Yes | 35 (21) | - | - |
| No data | 73 | - | - |
| **Deep white matter invasion** |  |  |  |
| No | 15 (9) | 1 | ref |
| Yes | 24 (13) | 1.17 (0.50-2.76) | 0.719 |
| No data | 73 | - | - |
| **Contrast enhancement quality** |  |  |  |
| None | 7 (4) | 1 | ref |
| Minimal/Mild | 17 (8) | 0.55 (0.16-1.90) | 0.342 |
| Marked/Avid | 12 (10) | 1.07 (0.32-3.55) | 0.916 |
| No data | 76 | - | - |
| **Contrast enhancement relationship to neural stem cell regions** |  |  |  |
| In the cortex and subependymal region | 8 (5) | 1 | ref |
| In the subependymal region, but not in the cortex | 8 (6) | 1.88 (0.56-6.34) | 0.310 |
| In the cortex, but not in the subependymal region | 12 (6) | 0.80 (0.24-2.63) | 0.709 |
| No data | 84 | - | - |
| **Contrast enhancement crossing the midline** |  |  |  |
| No | 25 (17) | - | - |
| Yes | 4 (1) | - | - |
| No data | 80 | - | - |
| **Non-contrast-enhanced tumor relationship to neural stem cell regions** |  |  |  |
| In the cortex and subependymal region | 15 (11) | 1 | ref |
| In the subependymal region, but not in the cortex | 4 (1) | - | - |
| In the cortex, but not in the subependymal region | 11 (5) | 0.62 (0.21-1.80) | 0.377 |
| Neither cortex nor ependymal involvement | 1 (1) | - | - |
| No data | 81 | - | - |
| **Non-contrast-enhanced tumor crosses midline** |  |  |  |
| No | 22 (14) | 1 | ref |
| Yes | 8 (3) | 0.55 (0.16-1.95) | 0.357 |
| No data | 81 | - | - |
| **T1/FLAIR ratio:** *defined as size of pre-contrast T1 abnormality (exclusive of signal intensity) versus size of FLAIR abnormality* |  |  |  |
| Expansive | 22 (12) | 1 | ref |
| Mixed | 7 (4) | 1.11 (0.35-3.50) | 0.859 |
| Infiltrative | 4 (4) | - | - |
| No data | 79 | - | - |
| **Diffusion** |  |  |  |
| Facilitated | 4 (4) | - | - |
| Restricted | 14 (8) | 1 | ref |
| Mixed | 11 (5) | 0.56 (0.17-1.87) | 0.344 |
| No data | 83 | - | - |
| **Necrosis** |  |  |  |
| No | 21 (13) | 1 | ref |
| Yes | 17 (9) | 0.41 (0.15-1.17) | 0.096 |
| No data | 74 | - | - |
| **Cyst** |  |  |  |
| Absent | 31 (20) | 1 | ref |
| Present | 8 (2) | 0.58 (0.13-2.51) | 0.463 |
| No data | 73 | - | - |
| **Hemorrhage** |  |  |  |
| No | 27 (13) | 1 | ref |
| Yes | 11 (8) | 1.78 (0.73-4.31) | 0.204 |
| No data | 74 | - | - |
| **Calvarial remodeling** |  |  |  |
| No | 32 (17) | 1 | ref |
| Yes | 7 (5) | 1.10 (0.40-3.01) | 0.853 |
| No data | 73 | - | - |

CI, confidence interval; HR, hazard ratio, n: number; ^a^, overall survival data are missing in 2 patients

Table S14. **Univariate analysis of type of H3 G34 mutation and histopathological markers for prognosis with regards to death in patients with diffuse hemispheric glioma, H3 G34-mutant (Cox regression) (n=112).^a^**

|  | **n (events)** | **HR (95% CI)** | **p-value** |
| --- | --- | --- | --- |
| **Type of G34 mutation** |  |  |  |
| G34R | 88 (53) | 0.71 (0.30-1.65) | 0.424 |
| G34V | 7 (6) | 1 | ref |
| G34M | 1 (1) | - | - |
| No data | 16 | - | - |
| **Cell density** |  |  |  |
| High | 59 (39) | 1.10 (0.61-2.00) | 0.750 |
| Moderate | 26 (16) | 1 | ref |
| Low | 2 (1) | - | - |
| No data | 25 | - | - |
| **Pleomorphism** |  |  |  |
| High | 12 (6) | 0.91 (0.22-3.70) | 0.893 |
| Moderate | 68 (47) | 1.23 (0.38-3.96) | 0.729 |
| Low | 7 (3) | 1 | ref |
| No data | 25 | - | - |
| **Differentiation** |  |  |  |
| Astrocytic | 61 (36) | 0.75 (0.43-1.31) | 0.316 |
| PNET or PNET-like component | 26 (20) | 1 | ref |
| No data | 25 | - | - |
| **Mitosis** |  |  |  |
| No | 6 (2) | 0.78 (0.18-3.39) | 0.742 |
| 1-9 | 30 (19) | 1 | ref |
| >10 | 36 (23) | 0.88 (0.48-1.62) | 0.683 |
| No data | 40 | - | - |
| **Necrosis** |  |  |  |
| Yes | 27 (19) | 1.25 (0.70-2.24) | 0.453 |
| Yes serpentine | 12 (9) | 1.47 (0.69-3.14) | 0.322 |
| No | 48 (28) | 1 | ref |
| No data | 25 | - | - |
| **Necrosis**  Yes, including sepentine  No  No data | 39 (28)  48 (28)  25 | 1.31 (0.78-2.22)  1  - | 0.312  ref  - |
| **Microvascular proliferation** |  |  |  |
| Yes | 51 (31) | 0.53 (0.31-0.91) | 0.020 |
| No | 36 (25) | 1 | ref |
| No data | 25 | - | - |
| **Multinuclear giant cells** |  |  |  |
| Yes | 9 (5) | 0.57 (0.22-1.45) | 0.236 |
| Yes, single | 21 (11) | 0.56 (0.29-1.10) | 0.094 |
| No | 57 (40) | 1 | ref |
| No data | 25 | - | - |
| **Calcification** |  |  |  |
| Yes | 6 (4) | 0.84 (0.30-2.33) | 0.735 |
| No | 81 (52) | 1 | - |
| No data | 25 | - | - |
| **Microvascular proliferation or necrosis** |  |  |  |
| Yes | 57 (36) | 0.62 (0.36-1.09) | 0.094 |
| No | 30 (20) | 1 | ref |
| No data | 25 | - | - |

CI, confidence interval; HR, hazard ratio, N: number; type of mutation remains unknown for tumors where diagnosis was based on methylation profiling (n=17); ^a^, overall survival data are missing in 2 patients.

Table S15. **Univariate analysis of molecular markers for prognosis with regards to death in patients with diffuse hemispheric glioma, H3 G34-mutant (Cox regression).**

|  | **n (events)** | **HR (95% CI)** | **p-value** |
| --- | --- | --- | --- |
| ***MGMT* promotor** |  |  |  |
| Unmethylated | 26 (20) | 1 | ref |
| Methylated | 77 (43) | 0.52 (0.30-0.89) | 0.018 |
| No data | 9 | - | - |
| **Focal copy number underrepresentation affecting the *MGMT* locus (cut-off 0.5)** |  |  |  |
| No | 59 (41) | 1 | ref |
| Yes | 21 (8) | 0.39 (0.18-0.85) | 0.017 |
| No data | 32 | - | - |
| **Focal copy number underrepresentation affecting the *MGMT* locus (cut-off 0.75)** |  |  |  |
| No | 69 (45) | 1 | ref |
| Yes | 11 (4) | 0.38 (0.14-1.07) | 0.066 |
| No data | 32 | - | - |
| **+ 7/-10 signature** |  |  |  |
| No | 66 (41) | 1 | ref |
| Yes | 14 (8) | 0.92 (0.43-1.99) | 0.839 |
| No data | 32 | - | - |
| **10q loss** |  |  |  |
| No | 63 (39) | 1 | ref |
| Yes | 17 (10) | 0.79 (0.39-1.59) | 0.507 |
| No data | 32 | - | - |
| ***CCND2* amplification** |  |  |  |
| No | 74 (46) | 1 | ref |
| Yes | 6 (3) | 0.50 (0.15-1.63) | 0.250 |
| No data | 32 | - | - |
| ***CDK6* amplification** |  |  |  |
| No | 68 (42) | 1 | ref |
| Yes | 12 (7) | 0.38 (0.17-0.87) | 0.022 |
| No data | 32 | - | - |
| ***EGFR* amplification** |  |  |  |
| No | 72 (44) | 1 | ref |
| Yes | 8 (5) | 0.70 (0.28-1.76) | 0.442 |
| No data | 32 | - |  |
| ***PDGFR* amplification** |  |  |  |
| No | 64 (38) | 1 | ref |
| Yes | 16 (11) | 1.12 (0.56-2.20) | 0.774 |
| No data | 32 | - | - |
| ***CDKN2A/B* homozygous deletion** |  |  |  |
| No | 64 (37) | 1 | ref |
| Yes | 16 (12) | 1.43 (0.74-2.76) | 0.289 |
| No data | 32 | - | - |
| ***RB* homozygous deletion** |  |  |  |
| No | 64 (39) | 1 | ref |
| Yes | 16 (10) | 1.48 (0.74-2.97) | 0.272 |
| No data | 32 | - | - |
| **Amplification in either *CCND1*, *CCND2*, *CDK4*, *CDK6*, *EGFR*, *PDGFR*, *MYCN*, or *MET*** |  |  |  |
| No | 52 (31) | 1 | ref |
| Yes | 28 (18) | 0.68 (0.37-1.25) | 0.213 |
| No data | 32 | - | - |

Survival data are missing in 2 patients with molecular markers available.

Table S16. **Patient characteristics stratified by *MGMT* promoter methylation and focal copy number underrepresentation affecting the *MGMT* locus (cut-off: <-0.5 (log2)).**

|  | ***MGMT* promoter methylated** | | ***MGMT* promoter unmethylated** | |
| --- | --- | --- | --- | --- |
|  | Focal copy number under-representation affecting the *MGMT* locus | No focal copy number under-representation affecting the *MGMT* locus | Focal copy number under-representation affecting the *MGMT* locus | No focal copy number under-representation affecting the *MGMT* locus |
|  | **n=16** | **n=45** | **n=5** | **n=15** |
| **Age at first surgery** |  |  |  |  |
| Median (years) | 25.5 | 22 | 20 | 27 |
| Interquartile interval (Q1-Q3) | 16-37 | 18-29 | 14-28 | 20-38 |
| p-value |  |  |  | 0.454 |
| **Sex, n (%)** |  |  |  |  |
| Male | 7 (44) | 30 (67) | 5 (100) | 12 (80) |
| Female | 9 (56) | 15 (33) | 0 (0) | 3 (20) |
| p-value |  |  |  | 0.058 |
| **KPS at diagnosis, n (%)** |  |  |  |  |
| 90-100% | 9 (69) | 20 (54) | 3 (60) | 7 (54) |
| 70-80% | 3 (23) | 12 (32) | 1 (20) | 3 (23) |
| <70% | 1 (8) | 5 (14) | 1 (20) | 3 (23) |
| No data | 3 (-) | 8 (-) | 0 (-) | 2 (-) |
| p-value |  |  |  | 0.899 |
| **Extent of resection, n (%)** |  |  |  |  |
| Gross total resection | 7 (47) | 17 (41) | 2 (40) | 6 (43) |
| Incomplete | 3 (20) | 11 (27) | 3 (60) | 8 (57) |
| Biopsy | 5 (33) | 13 (32) | 0 (0) | 0 (0) |
| No data | 1 (-) | 4 (-) | 0 (-) | 1 (-) |
| p-value |  |  |  | 0.089 |
| **First-line treatment, n (%)** |  |  |  |  |
| No therapy | 0 (0) | 2 (5) | 0 (0) | 3 (20) |
| Any therapy | 16 (100) | 41 (95) | 5 (100) | 12 (80) |
| Radiotherapy alone | 0 (0) | 2 (5) | 0 (0) | 1 (8) |
| Temozolomide alone | 0 (0) | 1 (2) | 0 (0) | 0 (0) |
| Temozolomide/radiotherapy |  | 1 (2) | 1 (20) | 2 (17) |
| Temozolomide/radiotherapy, followed by temozolomide | 13 (81) | 28 (68) | 3 (60) | 9 (75) |
| Temozolomide/radiotherapy, followed by temozolomide-based regimen | 2 (13) | 7 (17) | 1 (20) | 0 (0) |
| Radiotherapy followed by temozolomide | 0 (0) | 1 (2) | 0 (0) | 0 (0) |
| Surgery alone without adjuvant treatment | 0 (0) | 1 (2) | 0 (0) | 0 (0) |
| Other | 1^a^ (6) | 0 (0) | 0 (0) | 0 (0) |
| No data | 0 (-) | 2 (-) | 0 (-) | 0 (-) |
| p-value (treatment: yes versus no) |  |  |  | 0.096 |
| **Radiotherapy, n (%)** |  |  |  |  |
| Yes | 16 (100) | 39 (91) | 5 (100) | 12 (80) |
| No | 0 (0) | 4 (9) | 0 (0) | 3 (20) |
| No data | 0 (-) | 2 (-) | 0 (-) | 0 (-) |
| p-value |  |  |  | 0.225 |
| Information on dose, n (%)^b^ | 14 (88) | 33 (85) | 5 (100) | 12 (100) |
| Median dose | 60 | 60 | 60 | 60 |
| Interquartile interval (Q1-Q3) | 60-60 | 55-60 | 60-60 | 60-60 |
| **G34 mutation** |  |  |  |  |
| R | 13 (93) | 31 (82) | 5 (100) | 12 (92) |
| V | 1 (7) | 6 (16) | 0 (0) | 1 (8) |
| M | 0 (0) | 1 (3) | 0 (0) | 0 (0) |
| No data | 2 (-) | 7 (-) | 0 (-) | 3 (-) |
| p-value |  |  |  | 0.839 |

^a^, TMZ/RT-> unknown; ^b^, percentages calculated for the patients who had radiotherapy

Table S17. **Patient characteristics stratified by *MGMT* promoter methylation and focal copy number underrepresentation affecting the *MGMT* locus (cut-off < -0.75 (log2)).**

|  | ***MGMT* promoter methylated** | | ***MGMT* promoter unmethylated** | |
| --- | --- | --- | --- | --- |
|  | Focal copy number under-representation affecting the *MGMT* locus | No focal copy number under-representation affecting the *MGMT* locus | Focal copy number under-representation affecting the *MGMT* locus | No focal copy number under-representation affecting the *MGMT* locus |
|  | **n=9** | **n=52** | **n=2** | **n=18** |
| **Age at first surgery** |  |  |  |  |
| Median (years) | 24 | 23 | 17 | 25 |
| Interquartile interval (Q1-Q3) | 13-35 | 18-31 | 14-undefined | 20-35 |
| p-value^a^ |  |  |  | 0.529 |
| **Sex, n (%)** |  |  |  |  |
| Male | 4 (44) | 33 (64) | 2 (100) | 15 (83) |
| Female | 5 (56) | 19 (37) | 0 (0) | 3 (17) |
| p-value^b^ |  |  |  | 0.139 |
| **KPS at diagnosis, n (%)** |  |  |  |  |
| 90-100% | 4 (57) | 25 (58) | 2 (100) | 8 (50) |
| 70-80% | 2 (29) | 13 (30) | 0 (0) | 4 (25) |
| <70% | 1 (14) | 5 (12) | 0 (0) | 4 (25) |
| No data | 3 (-) | 8 (-) | 0 (-) | 2 (-) |
| p-value^b^ |  |  |  | 0.783 |
| **Extent of resection, n (%)** |  |  |  |  |
| Gross total resection | 4 (50) | 20 (42) | 1 (50) | 7 (41) |
| Incomplete | 1 (13) | 13 (27) | 1 (50) | 10 (59) |
| Biopsy | 3 (38) | 15 (31) | 0 (0) | 0 (0) |
| No data | 1 (-) | 4 (-) | 0 (-) | 1 (-) |
| p-value^b^ |  |  |  | 0.075 |
| **First-line treatment, n (%)** |  |  |  |  |
| No therapy | 0 (0) | 2 (4) | 0 (0) | 3 (17) |
| Any therapy | 9 (100) | 48 (96) | 2 (100) | 15 (83) |
| Radiotherapy alone | 0 (0) | 2 (4) | 0 (0) | 1 (7) |
| Temozolomide alone | 0 (0) | 1 (2) | 0 (0) | 0 (0) |
| Temozolomide/radiotherapy | 0 (0) | 1 (2) | 0 (0) | 3 (20) |
| Temozolomide/radiotherapy, followed by temozolomide | 8 (89) | 33 (69) | 2 (100) | 10 (67) |
| Temozolomide/radiotherapy, followed by temozolomide-based regimen | 0 (0) | 9 (19) | 0 (0) | 1 (7) |
| Radiotherapy followed by temozolomide | 0 (0) | 1 (2) | 0 (0) | 0 (0) |
| Surgery alone without adjuvant treatment | 0 (0) | 1 (2) | 0 (0) | 0 (0) |
| Other | 1* (11) | 0 (0) | 0 (0) | 0 (0) |
| No data | 0 (-) | 2 (-) | 0 (-) | 0 (-) |
| p-value (treatment: yes versus no)^b^ |  |  |  | 0.2172 |
| **Radiotherapy, n (%)** |  |  |  |  |
| Yes | 9 (100) | 46 (92) | 2 (100) | 15 (83) |
| No | 0 (0) | 4 (8) | 0 (0) | 3 (17) |
| No data | 0 (-) | 2 (-) | 0 (-) | 0 (-) |
| p-value^b^ |  |  |  | 0.480 |
| Information on dose, n (%)^i^ | 7 (78) | 40 (87) | 2 (100) | 15 (100) |
| Median dose | 60 | 60 | 60 | 60 |
| Interquartile interval (Q1-Q3) | 60-60 | 56-60 | 60-60 | 60-60 |
| **G34 mutation** |  |  |  |  |
| R | 7 (100) | 37 (82) | 2 (100) | 15 (94) |
| V | 0 (0) | 7 (16) | 0 (0) | 1 (6) |
| M | 0 (0) | 1 (2) | 0 (0) | 0 (0) |
| No data | 2 (-) | 7 (-) | 0 (-) | 2 (-) |
| p-value^b^ |  |  |  | 0.808 |

* TMZ/RT-> unknown; ^i^, percentages calculated for the patients who had radiotherapy; the Chi-square test was performed for the analysis of nominal variables^a^, and the independent-samples median test was used for the comparison of medians between groups^b^..

Table S18. **Prognostic associations (overall survival) of focal copy number underrepresentation affecting the *MGMT* locus.**

|  |  | **Underrepresentation of chromosome 10 including the *MGMT* locus** |  | **No underrepresentation of chromosome 10 including the *MGMT* locus** |  |
| --- | --- | --- | --- | --- | --- |
|  | n (%) | Median overall survival, (95% CI) Interquartile interval (Q1-Q3) | n (events) | Median overall survival, (95% CI) Interquartile interval (Q1-Q3) | P value |
|  |  |  |  |  |  |
| **CUT OFF: < -0.5 (log2)** |  |  |  |  |  |
| All patients | 21 (8) | 31.9  (0.0-97.2)  (22.8-not defined) | 59 (41) ^(a)^ | 19.4  (13.0-25.9)  (13.1-41.4) | 0.014 |
|  |  |  |  |  |  |
| *MGMT* promoter methylated | 16 (3) | not reached  not reached  (88.3-not defined) | 43 (30) | 18.5  (12.1-24.8)  (14.3-38.2) | 0.002 |
|  |  |  |  |  |  |
| *MGMT* promoter unmethylated | 5 (5) | 17.2  (0.0-36.7)  (8.1-26.4) | 15 (10) | 9.4  (0.0-29.8)  (5.8-67.9) | 0.733 |
|  |  |  |  |  |  |
| **CUT OFF < -0.75 (log2)** |  |  |  |  |  |
| All patients | 11 (4) | 88.3  (0.0-181.2)  (26.2-not defined) | 69 (45) ^(a)^ | 21.5  (14.8-28.2)  (13.6-41.4) | 0.057 |
|  |  |  |  |  |  |
| *MGMT* promoter methylated | 9 (2) | 88.3  (0.0-189.7)  (88.3-not defined) | 50 (31) | 21.5  (14.7-28.3)  (14.5-41.4) | 0.033 |
|  |  |  |  |  |  |
| *MGMT* promoter unmethylated | 2 (2) | 7.7  (not defined)  (7.9-26.4) | 18 (13) | 17.2  (1.6-32.7)  (8.1-31.9) | 0.756 |
|  |  |  |  |  |  |

(a) in the studied population of patients without underrepresentation of chromosome 10 including the *MGMT* locus, overall survival data are missing in 2 patients with a methylated MGMT promoter; the *MGMT* promoter methylation status was not determined in 1 case

Figure S1. **Identification of patients with H3 G34-mutant diffuse hemispheric gliomas locally at the participating centers.** Pie chart of technologies and their combination to assess the H3 G34 mutation status. A. Local diagnosis, B. After central review ^(1)^


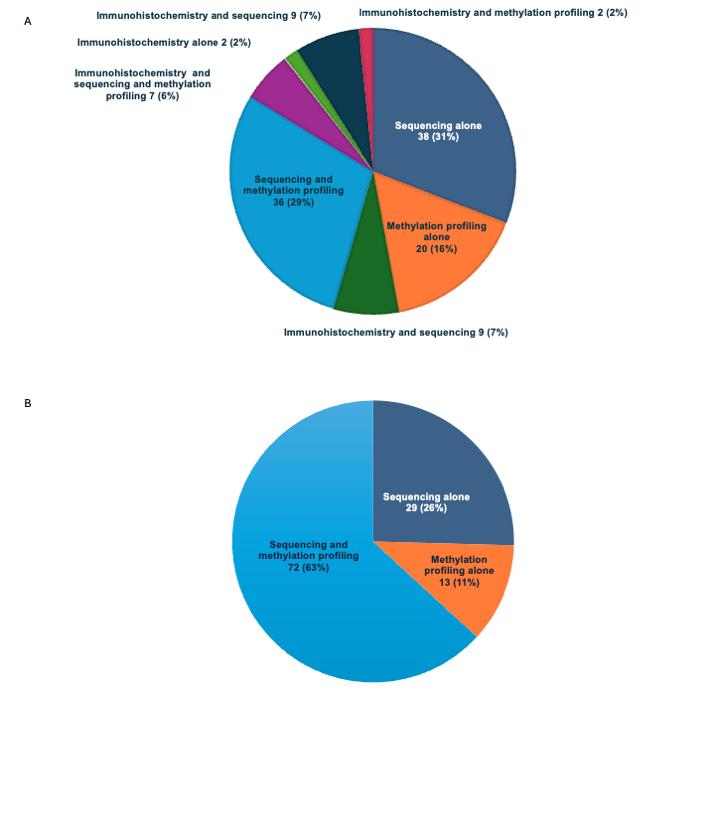


Numbers and percentage are provided.

(1): Sequencing alone include 26 cases centrally reviewed and 3 cases diagnosed locally. Methylation profiling alone include 12 cases centrally reviewed and 1 case diagnosed locally. The other 72 cases diagnosed by both sequencing and methylation profiling were all centrally reviewed.

Figure S2**.** **Illustration of histological features in H3 G34-mutant diffuse hemispheric gliomas.** Representative examples of hematoxylin-eosin (H&E) stained sections showing (A) low, (B) moderate, and (C) high cellularity; (D) low, (E) moderate. and (F) high cellular pleomorphism; (G) astrocytic differentiation, (H) mixed astrocytic and primitive neuroectodermal tumor (PNET)-like differentiation, and (I) PNET-like differentiation. Tumors variably showed (J) microvascular proliferation, (K) larger areas of necroses, and (L) serpentine necroses. M-O. Exemplary case of an H3 G34-mutant diffuse hemispheric glioma carrying an H3 p.G34R variant. (M) Histological appearance (H&E), (N) nuclear expression of H3 p.G34R, and (O) loss of nuclear ATRX expression. P-R. Exemplary case of an H3 G34-mutant diffuse hemispheric glioma carrying an H3 p.G34V variant. P Histological appearance (H&E), Q nuclear expression of H3 p.G34V, and R loss of nuclear ATRX expression Scale bars: 50 µm.


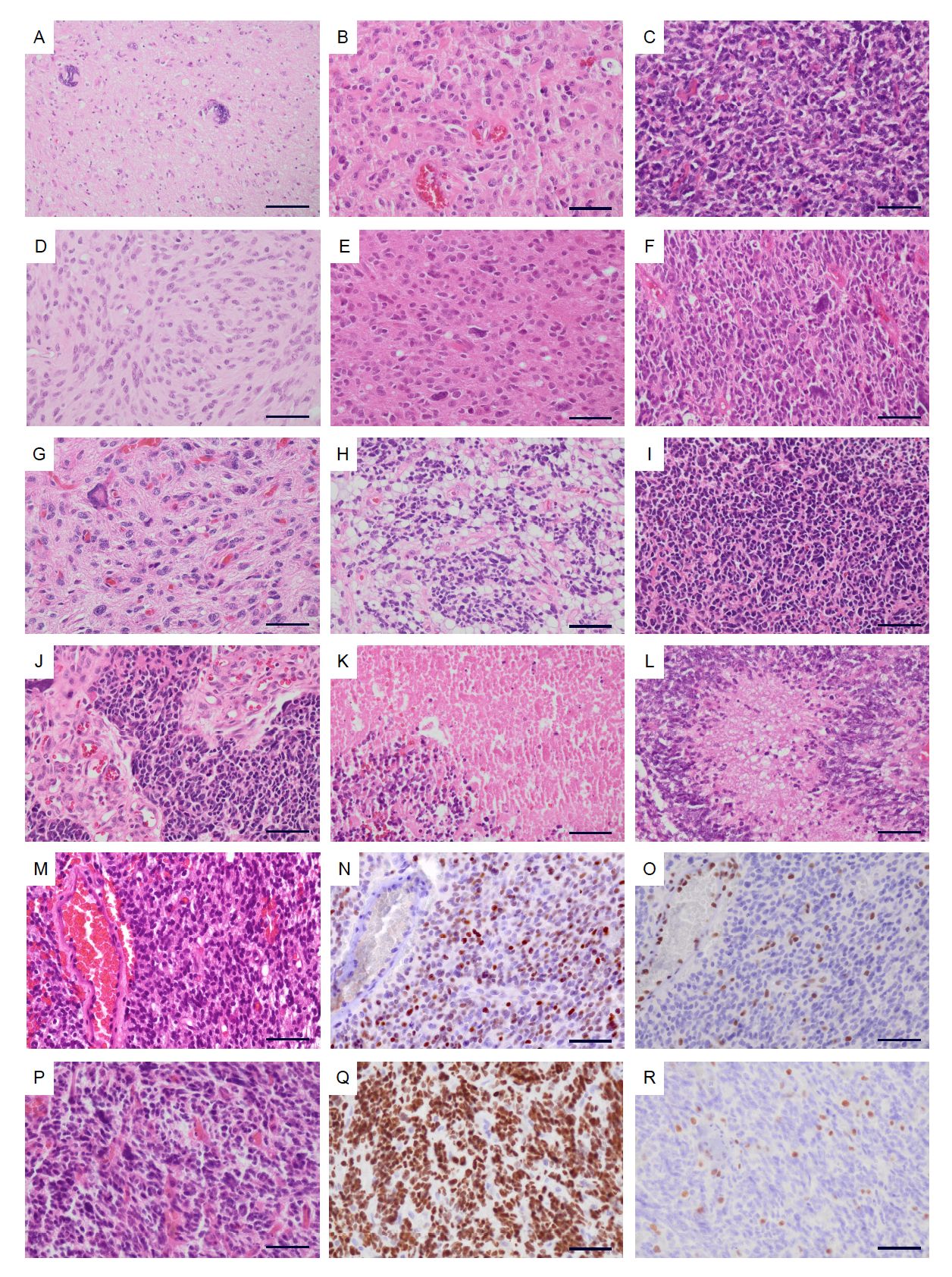


Figure S3. **tSNE plot and CNV profiles: H3 G34R- vs H3 G34V-mutant diffuse hemispheric gliomas.**

|  | Tumors (n) |
| --- | --- |
| Total | 84 |
| H3 G34M | 1 |
| H3 G34R | 63 |
| H3 G34V | 8 |
| Sequenced no mutation | 2 |
| No data | 10 |

Figure S4. **tSNE plot and CNV profiles: tumors of female versus male patients with diffuse hemispheric gliomas, H3 G34-mutant.** For the annotations of the reference cohorts, see Figure S3.

|  | Patients (n) |
| --- | --- |
| Total | 84 |
| Males | 57 |
| Females | 27 |

Figure S5. **tSNE plot and CNV profiles: age associations in patients with diffuse hemispheric gliomas, H3 G34-mutant.** Three groups of young, intermediate and older patients were defined based on the factual range of age at diagnosis in the present cohort in quartiles. For the annotations of the reference cohorts, see Figure S3.

|  | Patients (n) |
| --- | --- |
| Total | 84 |
| Young age group (17 years or less) | 18 |
| Medium age group (18-30 years) | 44 |
| Old age group (31 or more years) | 22 |

Figure S6. **tSNE plot and CNV profiles: associations with tumor location and extension in patients with diffuse hemispheric gliomas, H3 G34-mutant.** For the annotations of the reference cohorts, see Figure S3.

|  | Tumors (n) |
| --- | --- |
| Total | 84 |
| Frontal | 22 |
| Temporal | 20 |
| Parietal | 19 |
| Occipital | 5 |
| More than 1 lobe | 18 |

Figure S7. **tSNE plot and CNV profiles: associations with contrast enhancement by central MRI review in patients with diffuse hemispheric gliomas, H3 G34-mutant.** For the annotations of the reference cohorts, see Figure S3.

|  | Tumors (n) |
| --- | --- |
| Total | 33 |
| None/minimal/mild | 18 |
| Marked/avid | 12 |
| No data | 3 |

Figure S8. **tSNE plot and CNV profiles: associations with diffusion restriction by central MRI review in patients with diffuse hemispheric gliomas, H3 G34-mutant.** For the annotations of the reference cohorts, see Figure S3.

|  | Tumors (n) |
| --- | --- |
| Total | 33 |
| Facilitated | 4 |
| Mixed | 10 |
| Restricted | 12 |
| No data | 7 |

Figure S9. **tSNE plot and CNV profiles: associations with detection of necrosis by central MRI review in patients with diffuse hemispheric gliomas, H3 G34-mutant.** For the annotations of the reference cohorts, see Figure S3.

|  | Tumors (n) |
| --- | --- |
| Total | 33 |
| Necrosis | 15 |
| No necrosis | 18 |
| No data | 7 |

Figure S10. **tSNE plot and CNV profiles: associations with astrocytic versus PNET-like histology by central pathological review in patients with diffuse hemispheric gliomas, H3 G34-mutant.** For the annotations of the reference cohorts, see Figure S3.

|  | Tumors (n) |
| --- | --- |
| Total | 71 |
| PNET | 22 |
| Astrocytic | 49 |

Figure S11. **tSNE plot and CNV profiles: associations with histological detection of necrosis.** For the annotations of the reference cohorts, see Figure S3.

|  | Tumors (n) |
| --- | --- |
| Total | 71 |
| Necrosis, serpentine | 11 |
| Necrosis | 22 |
| No necrosis | 38 |

Figure S12. **Diffuse hemispheric glioma, H3 G34-mutant: *MGMT* promoter methylation and focal copy number underrepresentation affecting the *MGMT* locus.** A. tSNE showing clustering of tumors with *MGMT* loss. B. Representative CNV of three selected tumors with focal copy number underrepresentation affecting the *MGMT* locus (arrows).

**A**

~~~~

~~
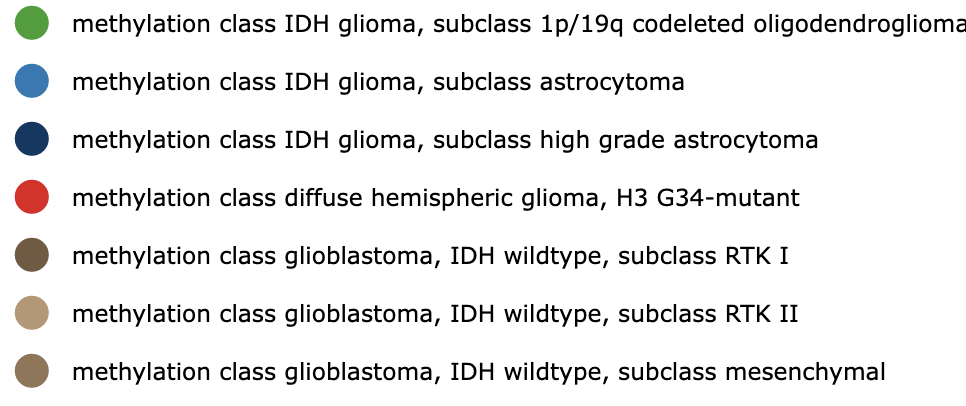
~~

blue dots in G34-mutant tumors: *MGMT* gene loss, *MGMT* promoter methylated

yellow dots in G34-mutant tumors: *MGMT* gene loss, *MGMT* promoter unmethylated

**B**

Figure S13. **DNA variants detected by gene panel sequencing in H3 G34R- versus H3 G34V-mutant diffuse hemispheric gliomas.**


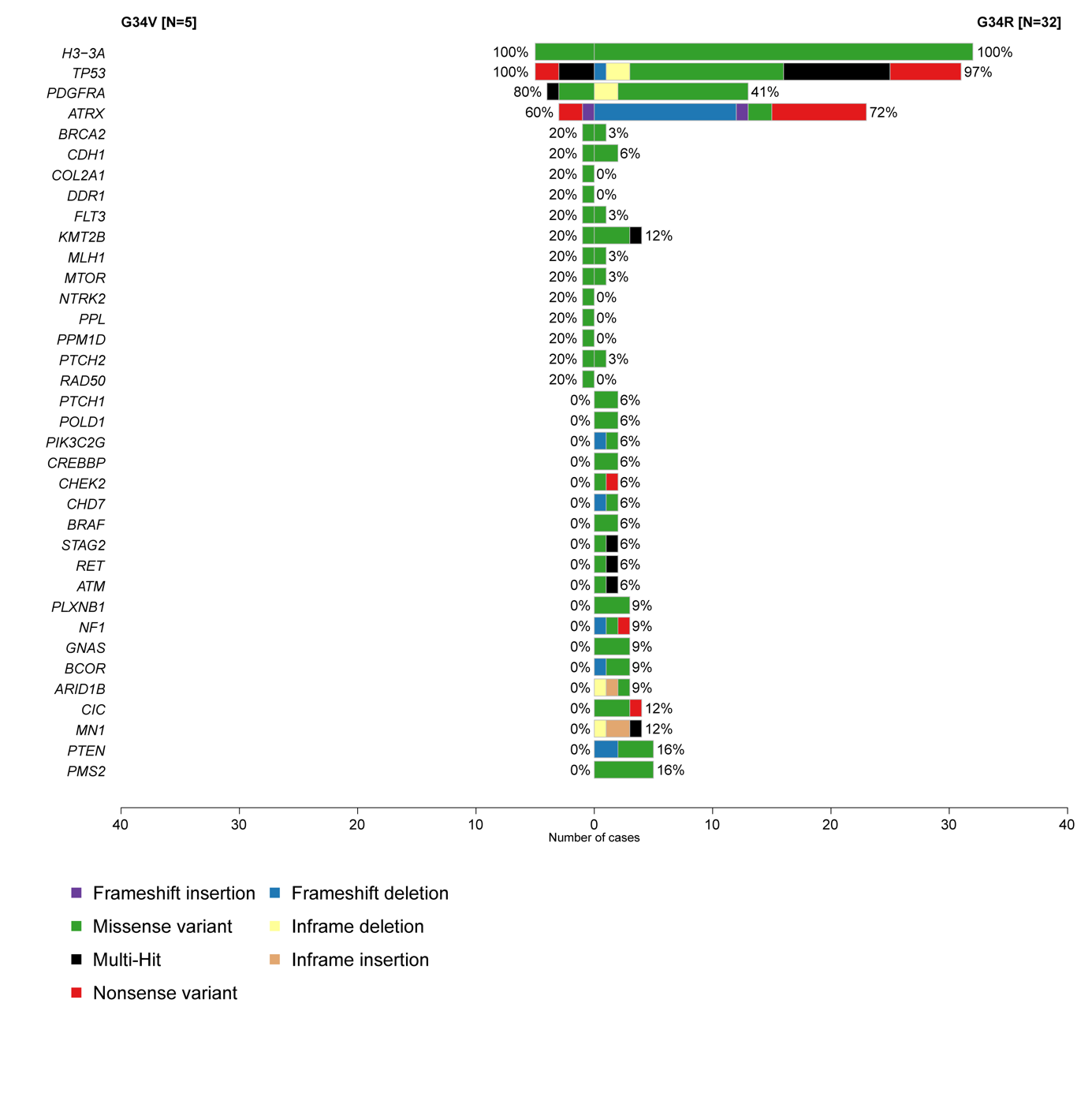


Figure S14. ***TP53* mutations in diffuse hemispheric glioma, H3 G34-mutant versus other gliomas: schematic p53 protein structure and variant location.** *TP53* mutation profiles in the investigated cohort, compared with H3 K27M-altered diffuse midline glioma and IDH-wildtype glioblastoma subtypes (MES, RTK1, RTK2), were visualized using ProteinPaint ^4^

Figure S15. **tSNE plot and CNV profiles: associations with long term survivors in patients with diffuse hemispheric gliomas, H3 G34-mutant.** For the annotations of the reference cohorts, see Figure S3.

|  | Patients (n) |
| --- | --- |
| Survival event more than 5 years | 8 |
| Survival event less than 5 years | 47 |
| No information on follow-up | 29 |

Figure S16. **tSNE plot and CNV profiles: associations with survival in patients with diffuse hemispheric gliomas, H3 G34-mutant.** Three groups of short, intermediate and long survival were defined based on the factual range of survival (quartiles) in the present cohort. For the annotations of the reference cohorts, see Figure S3.

|  | Patients (n) |
| --- | --- |
| Total | 84 |
| Short survival | 21 |
| Intermediate survival | 41 |
| Long survival | 20 |
| No data | 2 |

Figure S17. **tSNE plot and CNV profiles: associations with survival restricted to deceased patients with diffuse hemispheric gliomas, H3 G34-mutant.** For the annotations of the reference cohorts, see Figure S3.

|  | Number of cases |
| --- | --- |
| Total | 48 |
| Short survival | 12 |
| Intermediate survival | 22 |
| Long survival | 14 |
| No data | 2 |

Note S1. **Reference cohorts.**

The observations made in the primary study population of patients with diffuse hemispheric glioma, H3 G34-mutant were placed in perspective to other patient cohorts, depending on the specific question and dataset. A cohort of glioblastomas, IDH-wildtype, from the Canton of Zurich was used to generate “reference cohorts” 1 (Table 1, Figure 4) and 2 (Figure 1, Table S2) for some specific research questions, including comparisons of outcome.^5,6^ In reference cohort 1, tumors from patients aged younger than 55 years at diagnosis underwent sequencing for rare IDH mutations if IDH1R132H immunohistochemistry was negative.^7^ One hundred fifty-three patients aged 55 or more had the IDH status assessed by immunohistochemistry only. Reference cohort 1 was not specifically analyzed for H3 G34 mutations, however, one patient with known H3 G34-mutant tumor was excluded. Reference cohort 3 corresponds to patients of the German Glioma Network (Figure 2, Tables S5, S6). Reference cohort 4 stems from the recent study of Reuss et al.^3^ For reference cohorts 5 (Figure 3B) and 6 (Figure S14), data from the molecular database of the Department of Neuropathology in Heidelberg were used for which DNA methylation and sequencing data were available.

Note S2. **Extended information on gene panel sequencing.**

Library preparation adhered to the manufacturer's recommendations, employing the SureSelect XT HS2 DNA Kit (Agilent, #5191-5688). The libraries were pooled at 1.5 nM and subjected to paired-end sequencing on the Illumina Novseq6000 platform (Novaseq v1.5 200 cycles S1 Reagent Kit, #20028318). The NGS panel is tailored to encompass the entire coding region (all exons + /– 50 bp) along with selected intronic and promoter regions of at least 130 genes (depending on version) specifically pertinent to CNS tumors. Its design enables the detection of single nucleotide variants (SNV), small insertions/deletions (InDels), exonic rearrangements, and recurrent fusion events. Sequenced reads were mapped to GRCh38 using the nf-core/sarek (v3.3.2) pipeline. SNV and structural variant calling was done using Strelka (v4.4.0.0) and Manta (v1.6.0). Annotation of the detected variants was performed using SNPeff (v5.1d). All variants were filtered using the nf-core/sarek pipeline filters and additionally: map to exonic regions, phred-scaled probability (QUAL) > 20, mean of the mapping quality of reads (MQ) > 30, read depth (DP) > 15, high/moderate impact, and a population frequency < 0.001 based on the 1000G project.

Note S3. **Associations of histological differentiation with *PDGFR* or *CCND2* amplification.**

Among patients with central pathology review, 10 of 47 patients (21%) with tumors with astrocytic differentiation had *PDGFR* amplification, compared with 4 of 22 patients (18%) with tumors with PNET-like morphology (p=0.766). Further, *CCND2* amplification was seen in 4 of 47 patients (9%) with tumors with astrocytic differentiation compared with 1 of 28 patients (5%) with tumors with PNET-like morphology (p=0.554).

**References**

1. Nestler U, Lutz K, Pichlmeier U, et al. Anatomic features of glioblastoma and their potential impact on survival. *Acta Neurochir (Wien)*. 2015;157(2):179-186. doi:10.1007/s00701-014-2271-x

2. Lim DA, Cha S, Mayo MC, et al. Relationship of glioblastoma multiforme to neural stem cell regions predicts invasive and multifocal tumor phenotype. *Neuro Oncol*. 2007;9(4):424-429. doi:10.1215/15228517-2007-023

3. Reuss DE, Schrimpf D, Cherkezov A, et al. Heterogeneity of DNA methylation profiles and copy number alterations in 10782 adult-type glioblastomas, IDH-wildtype. *Free Neuropathol*. 2024;5:5-7. doi:10.17879/freeneuropathology-2024-5345

4. Zhou X, Edmonson MN, Wilkinson MR, et al. Exploring genomic alteration in pediatric cancer using ProteinPaint. *Nat Genet*. 2016;48(1):4-6. doi:10.1038/ng.3466

5. Gramatzki D, Dehler S, Rushing EJ, et al. Glioblastoma in the Canton of Zurich, Switzerland revisited: 2005 to 2009. *Cancer*. 2016;122(14):2206-2215. doi:10.1002/cncr.30023

6. Gramatzki D, Roth P, Rushing EJ, et al. Bevacizumab may improve quality of life, but not overall survival in glioblastoma: an epidemiological study. *Ann Oncol*. 2018;29(6):1431-1436. doi:10.1093/annonc/mdy106

7. Louis DN, Perry A, Wesseling P, et al. The 2021 WHO Classification of Tumors of the Central Nervous System: a summary. *Neuro Oncol*. 2021;23(8):1231-1251. doi:10.1093/neuonc/noab106
